# Constitutively active BRS3 is a genuinely orphan GPCR in placental mammals

**DOI:** 10.1371/journal.pbio.3000175

**Published:** 2019-03-06

**Authors:** Huihao Tang, Chuanjun Shu, Haidi Chen, Xiaojing Zhang, Zhuqing Zang, Cheng Deng

**Affiliations:** 1 Jiangsu Key Laboratory for Biodiversity and Biotechnology, College of Life Sciences, Nanjing Normal University, Nanjing, China; 2 Department of Bioinformatics, College of Biomedical Engineering and Information, Nanjing Medical University, Nanjing, China; University of Michigan, UNITED STATES

## Abstract

G protein–coupled receptors (GPCRs) play an important role in physiology and disease and represent the most productive drug targets. Orphan GPCRs, with their endogenous ligands unknown, were considered a source of drug targets and consequently attract great interest to identify their endogenous cognate ligands for deorphanization. However, a contrary view to the ubiquitous existence of endogenous ligands for every GPCR is that there might be a significant overlooked fraction of orphan GPCRs that function constitutively in a ligand-independent manner only. Here, we investigated the evolution of the bombesin receptor–ligand family in vertebrates in which one member—bombesin receptor subtype-3 (BRS3)—is a potential orphan GPCR. With analysis of 17 vertebrate BRS3 structures and 10 vertebrate BRS3 functional data, our results demonstrated that nonplacental vertebrate BRS3 still connects to the original ligands—neuromedin B (NMB) and gastrin-releasing peptide (GRP)—because of adaptive evolution, with significantly changed protein structure, especially in three altered key residues (Q127R, P205S, and R294H) originally involved in ligand binding/activation, whereas the placental mammalian BRS3 lost the binding affinity to NMB/GRP and constitutively activates Gs/Gq/G12 signaling in a ligand-independent manner. Moreover, the N terminus of placental mammalian BRS3 underwent positive selection, exhibiting significant structural differences compared to nonplacental vertebrate BRS3, and this domain plays an important role in constitutive activity of placental mammalian BRS3. In conclusion, constitutively active BRS3 is a genuinely orphan GPCR in placental mammals, including human. To our knowledge, this study identified the first example that might represent a new group of genuinely orphan GPCRs that will never be deorphanized by the discovery of a natural ligand and provided new perspectives in addition to the current ligand-driven GPCR deorphanization.

## Abstract 2

“Orphan” G protein–coupled receptors (GPCRs) are membrane-bound receptors for which a natural ligand has yet to be identified. This study shows that the mammalian GPCR BRS3 represents a new group of GPCRs that will never be deorphanized, as they function constitutively in a ligand-independent manner.

## Author summary

The current model on G protein–coupled receptors (GPCRs) is that they have at least one endogenous ligand for the activation of the heterotrimeric G proteins that controls lots of physiological functions. Currently, 140 out of approximately 800 GPCRs are referred to as orphan because of a lack of knowledge about their endogenous ligands, and they attract great interest in major medical institutes and pharmaceutical companies. Can a “ligand-receptor” model apply to all the GPCRs? Our study shows that it probably cannot. By studying the evolution of a classic orphan GPCR–bombesin receptor subtype-3 (BRS3), we show that placental mammalian BRS3 is constitutively active, in contrast to nonplacental vertebrate BRS3, which actually connects to its original ligands—neuromedin B (NMB) and gastrin-releasing peptide (GRP). Protein structure analysis and experiment data also suggest that placental mammalian BRS3, including human BRS3, lost connection with its original ligands during placental mammalian evolution. Therefore, we propose a new point of view that a considerable number of orphan GPCRs do not have endogenous ligands and might represent a new group of GPCRs that are genuinely orphan GPCRs and will never be deorphanized by discovery of a natural ligand. This new finding will provide new perspectives in current ligand-driven GPCR deorphanization.

## Introduction

G protein–coupled receptors (GPCRs), also known as seven-transmembrane (7TM) receptors, represent the largest superfamily of more than 800 vertebrate transmembrane proteins, and the main characteristic feature of these proteins is that they share a common 7TM configuration [1, 2]. GPCRs have attracted great interest owing to their numerous physiological and pathological roles in transducing extracellular signals into intracellular effector pathways through the activation of heterotrimeric G protein (e.g., Gs, Gi, Gq, and G12) by binding to a broad range of ligands, including proteins, peptides, eicosanoids, and small organic compounds [1, 3]. Moreover, in humans, GPCRs have been proven to be the most successful class of drug targets, and 30%–50% of marketed drugs are estimated to exert their clinical effects via GPCRs [4]. Therefore, more than 140 GPCRs—named orphan GPCRs, which were considered to have endogenous ligands—are potential therapeutic targets, and they attracted a great deal of interest in the search for their endogenous ligands for deorphanization [3–8].

Bombesin (also called BBS or BN) is a tetradecapeptide originally isolated from the skin of the European fire-bellied toad *Bombina bombina* and only found in amphibians [9]. Two bombesin-like peptides (BLPs), gastrin-releasing peptide (GRP) and neuromedin B (NMB), were found to be conserved across vertebrates. The biological actions of NMB and GRP are mediated via their specific receptors, namely NMB receptor (NMBR, BB1) and GRP receptor (GRPR, BB2), respectively [10]. NMBR/GRPR stimulation of intracellular calcium and extracellular signal–regulated kinase (ERK) require their cognate peptide ligands via Gq signaling [1, 11]. But neither NMB nor GRP is the cognate ligand for the third bombesin receptor, named bombesin receptor subtype-3 (BRS3, BB3), although it shows high sequence identity to GRPR (approximately 47%) and NMBR (approximately 44%). BLPs exhibit a large degree of sequence conservation across most vertebrates [12]. Therefore, BRS3 was considered as an orphan Gq-coupled GPCR, and some studies proposed the existence of a BRS3 cognate peptide hormone, which should be a natural and endogenous BLP [11–13]. Also, many synthetic ligands were designed for BRS3, even with high binding affinity [14]. However, thus far, the natural peptide ligand for BRS3 has not been identified, and BRS3 is still considered as an orphan GPCR [15].

Almost every GPCR is presumed to interact with endogenous cognate ligand(s) in our body, and therefore orphan GPCRs attract a great deal of interest in search for endogenous ligands, eventually leading to deorphanization [7, 16]. On the other hand, some studies demonstrated that orphan GPCRs can function in a ligand-independent manner—e.g., constitutively activating the G protein signaling—by heterodimerizing with other GPCRs and functioning as a co-receptor [17–20]. Instead of postulating a natural ligand for each GPCR, namely constitutive functions for some orphan GPCRs have not been much illuminated [21, 22]. Here, with analysis of 17 vertebrate BRS3 structures and 10 vertebrate BRS3 functional data, we showed that BRS3, as a classic orphan GPCR originating from an ancestor of NMBR and GRPR, still connects to its original ligands NMB and GRP in nonplacental vertebrates but not in placental mammals. However, placental mammalian BRS3 underwent positive selection, and in comparison to nonplacental vertebrate BRS3, its protein structure is altered significantly. With three key residues (R127Q, S205P, and H294R) that regulate ligand binding/activation altered, placental mammalian BRS3 lost binding affinity to NMB/GRP and, because of additional changes in the N-terminal domain and G protein selectivity barcodes, constitutively activates Gs, Gq, and G12 signaling in a ligand-independent manner. Our study identified the first example, to our knowledge, that constitutively active BRS3 is a genuinely orphan GPCR in placental mammals, including humans.

## Results

### Evolution of the bombesin receptor–ligand family

**Fig 1:**
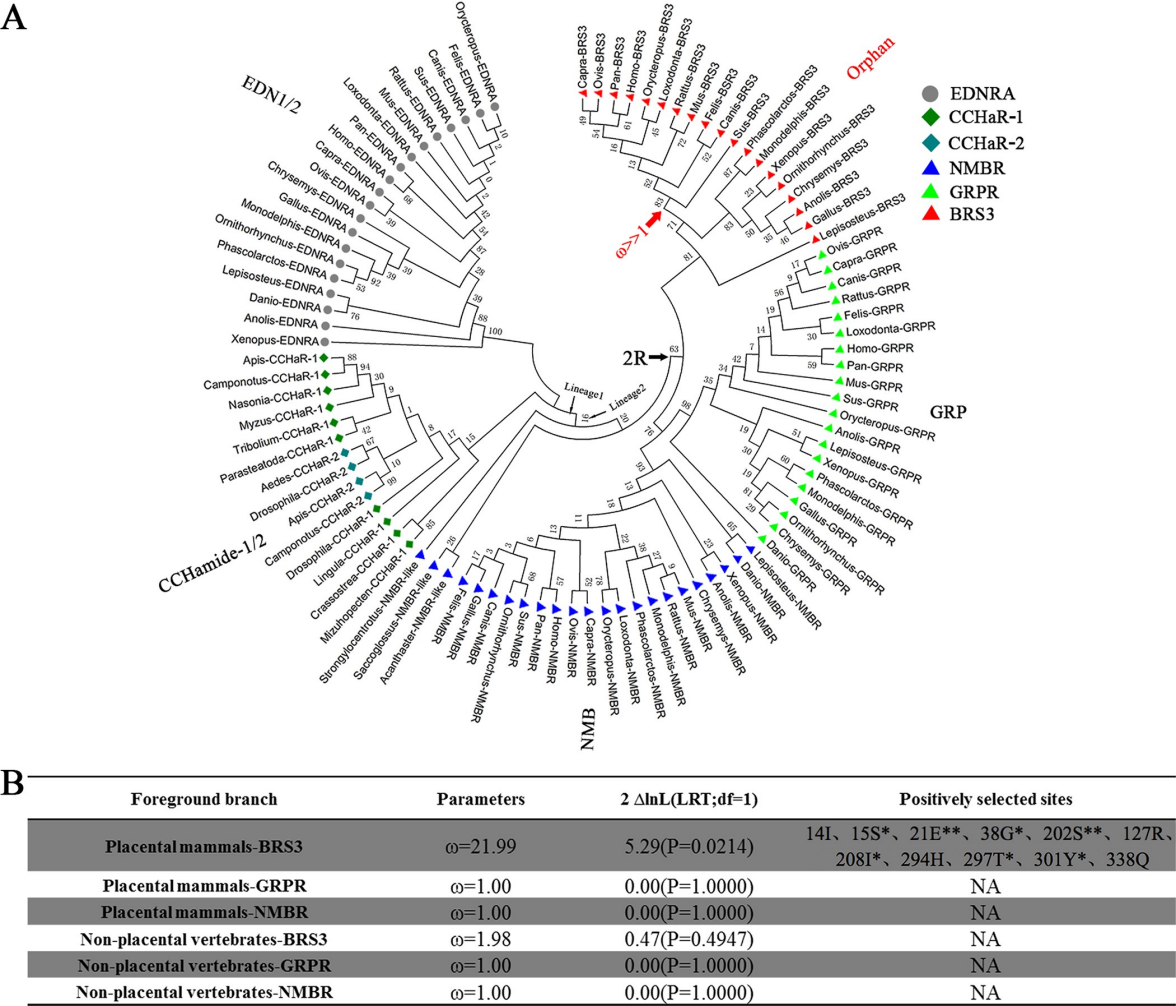
The evolution of the bombesin receptors. (A) Phylogenetic tree of bombesin receptors. The ligands were shown in vicinity to their corresponding receptors. The ligand for BRS3 remained elusive thus far. The species of phylogenetic tree are as follows: *Homo*: *Homo sapiens*; *Pan*: *Pan troglodytes*; *Mus*: *Mus musculus*; *Rattus*: *Rattus norvegicus*; *Sus*: *Sus scrofa*; *Capra*: *Capra hircus*; *Ovis*: *Ovis aries*; *Canis*: *Canis lupus familiaris*; *Felis*: *Felis catus*; *Orycteropus*: *Orycteropus afer*; *Loxodonta*: *Loxodonta africana*; *Phascolarctos*: *Phascolarctos cinereus*; *Monodelphis*: *Monodelphis domestica*; *Ornithorhynchus*: *Ornithorhynchus anatinus*; *Gallus*: *Gallus gallus*; *Anolis*: *Anolis carolinensis*; *Chrysemys*: *Chrysemys picta*; *Xenopus*: *Xenopus tropicalis*; *Lepisosteus*: *Lepisosteus oculatus*; *Danio*: *Danio rerio*; *Saccoglossus*: *Saccoglossus kowalevskii*; *Acanthaster*: *Acanthaster planci*; *Strongylocentrotus*: *Strongylocentrotus purpuratus*; *Apis*: *Apis mellifera*; *Nasonia*: *Nasonia vitripennis*; *Drosophila*: *Drosophila melanogaster*; *Aedes*: *Aedes aegypti*; *Tribolium*: *Tribolium castaneum*; *Camponotus*: *Camponotus floridanus*; *Parasteatoda*: *Parasteatoda tepidariorum*; *Myzus*: *Myzus persicae*; *Lingula*: *Lingula anatina*; *Crassostrea*: *Crassostrea virginica*; *Mizuhopecten*: *Mizuhopecten yessoensis*. (B) The parameters and statistical significance of LRTs for each branch of BRS3, GRPR, and NMBR are given; the dN/dS ratio is calculated with the whole protein coding region; **, *, and no * indicate *P* values in excess of 0.99, 0.95, and 0.90, respectively. 2R, two rounds of whole-genome duplications; BRS3, bombesin receptor subtype-3; CCHaR-1, CCHamide-1 receptor; CCHaR-2, CCHamide-2 receptor; df, degree of freedom; EDNRA, endothelin receptor type A; GRP, gastrin-releasing peptide; GRPR, GRP receptor; LRT, likelihood ratio test; NA, not available; NMBR, neuromedin B receptor.

To explore the origin of BRS3, the genetic relationship among BRS3 receptors, GRPRs, and NMBRs (the latter two having >44% sequence identity to BRS3), as well as CCHamide-1 receptor (CCHaR-1) and CCHamide-2 receptor (CCHaR-2), which also belong to the BRS3 phylogenetic subgroup, was included. The endothelin receptor type A (EDNRA) was used as an out-group, since it has low amino acid sequence identity (above 25%) relative to BRS3. The corresponding vertebrate amino acid sequences (*n* = 96) were downloaded from NCBI and Ensemble databases to reveal the evolutionary processes within the bombesin receptor family (S1 Table). A consensus tree was then built using MEGA 7.0.26 (JTT+G+I, bootstrap = 500, cutoff for condensed tree = 20%) (Fig 1A). As shown in Fig 1A, BRS3, GRPR, and NMBR were present in vertebrates such as fishes, amphibians, reptiles, birds, and mammals. However, NMBR-like members were also detected in a few nonvertebrate deuterostomes (such as Hemichordata and Echinodermata). Nonvertebrate deuterostome receptors may belong to prevertebrate NMBR/GRPR/BRS3 members, and vertebrate receptors are just NMBR but not NMBR-like members. In contrast, CCHaR-1 and CCHaR-2 receptors were only detectable in protostomes, such as Arthropoda, Brachiopoda, and Mollusca (Fig 1A).

Two lineages were shown in Fig 1A, one representing protostome receptors (Lineage 1) and the other representing deuterostome receptors (Lineage 2). Prior to the two rounds of whole-genome duplications (2R) [23], mainly deuterostome NMBR-like was present in lineage 2, indicating that bombesin receptors of vertebrates originated from the deuterostome NMBR-like genes. After the 2R event during the origin of vertebrates, a NMBR/GRPR/BRS3 progenitor expanded to three receptor subtypes: NMBR pairs with its endogenous peptide, NMB; GRPR pairs with its endogenous peptide, GRP; BRS3 has been considered an orphan GPCR since its 1980 identification [11, 24, 25]. The protein products of two paralogues, CCHaR-1 and CCHaR-2, share high amino acid similarity (above 80%) in amino acid sequences, binding two highly similar (>80%) endogenous peptides CCHamide-1 and CCHamide-2, respectively. This indicates an independent duplication of both receptor and ligand genes in protostomes (Fig 1A) [26]. The phylogenetic tree also suggested that BRS3 originated from a common ancestor of NMBR and GRPR and might still bind the endogenous ligands NMB and/or GRP in early stages of vertebrate origination.

To assess whether the bombesin receptors underwent positive selection after the 2R event, a branch-site model was utilized. As shown in Fig 1B, the result showed that only in placental mammal lineage, BRS3 sequences have a large nonsynonymous (dN)/synonymous (dS) substitution rate ratio (branch-site dN/dS of ω >> 1; Fig 1A) that is highly significant (likelihood ratio tests [LRTs], *P* < 0.05; Fig 1B). In contrast, all other vertebrates do not exhibit this ratio, suggesting that positive Darwinian selection occurred specifically in placental mammalian BRS3 but not in nonplacental vertebrates, including Marsupialia and Monotremata (Fig 1A and 1B). In contrast, no positive selection was detected in GRPR and NMBR (Fig 1B). In conclusion, BRS3 originated from the NMBR-like gene after the 2R event and underwent adaptive selection in placental mammals.

### Different evolution, different protein structures, and different function between nonplacental vertebrate BRS3 and placental mammalian BRS3: NMB and GRP are the endogenous cognate ligands for BRS3 in nonplacental vertebrates, whereas BRS3 in placental mammals constitutively activate Gs, Gq, and G12 signaling

**Fig 2:**
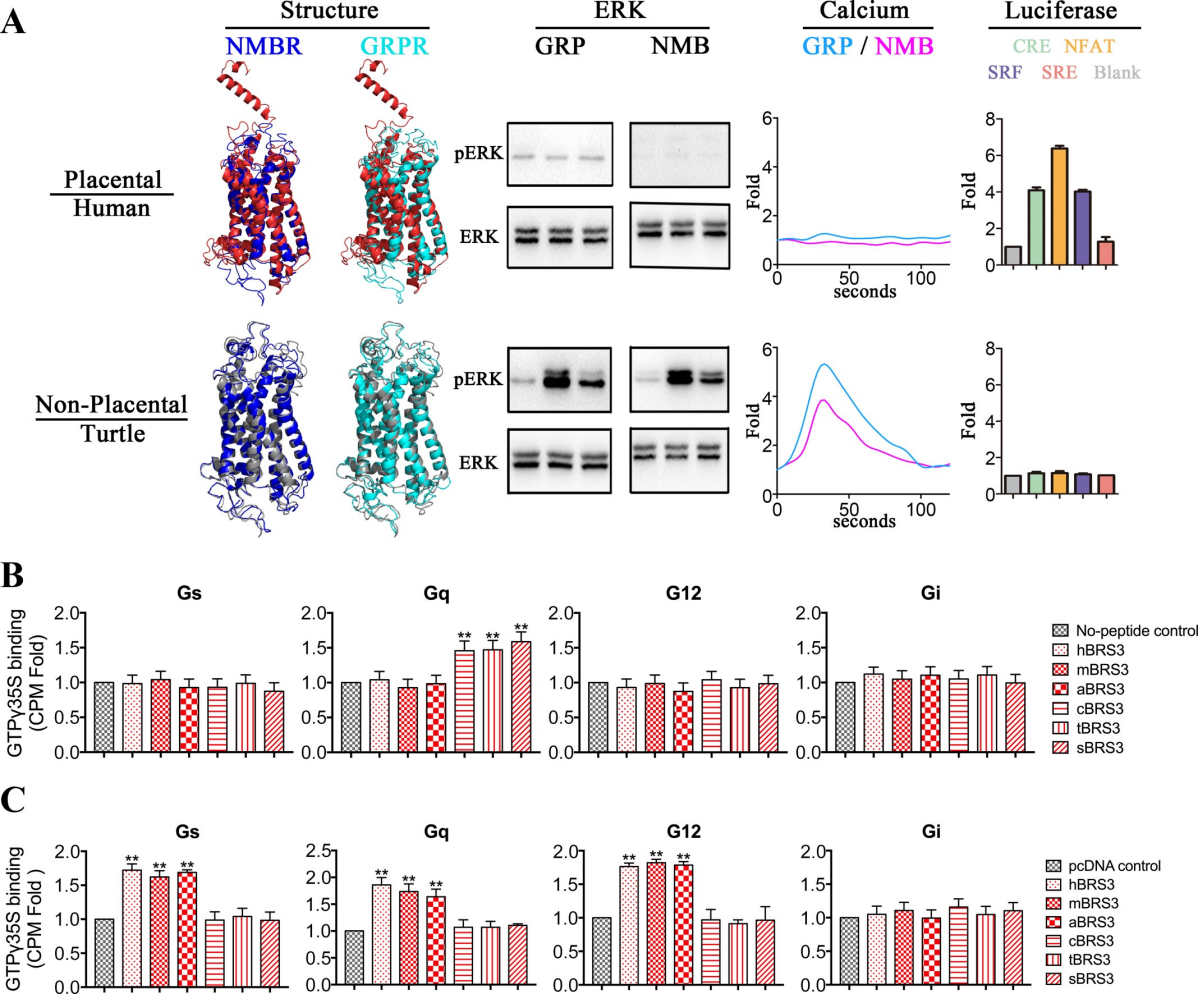
Representation of different protein structures and different function between nonplacental vertebrate BRS3 and placental mammalian BRS3. (A) Two representative species are shown: human represents placental mammals, and turtle represents nonplacental vertebrates. Four structure comparisons were predicted as representation: red, gray, cyan, and blue represent placental mammalian BRS3, nonplacental vertebrate BRS3, NMBR, and GRPR, respectively. The ERK lane: the phosphorylation levels of ERK for each of BRS3 receptors. GRP and NMB peptides are utilized to activate BRS3 in placental mammals and nonplacental vertebrates, respectively. Three time points of 0, 2, and 5 min were chosen. The calcium lane: the levels of Ca^2+^ ions in cells for each of the BRS3 receptors. The calcium fold is calculated by fluorescence intensity (excitation/emission wavelength: 490/520 nm). The luciferase lane: constitutive activity for BRS3 in placental mammals but not in nonplacental vertebrates. The luciferase fold is calculated by luminescence intensity. (B) Nonplacental BRS3 coupled with Gq in a ligand-dependent manner with GTPgamma35S incorporation assay. The results represent mean ± SEM of raw data from three independent experiments performed in triplicates. (C) Placental mammalian BRS3 coupled with Gs, Gq, and G12 in a ligand-independent manner with GTPgamma35S incorporation assay. The underlying data can be found in S1 Data. aBRS3, aardvark BRS3; BRS3, bombesin receptor subtype-3; cBRS3, chicken BRS3; CPM, counts per minute; CRE, cAMP response element; ERK, extracellular signal–regulated kinase; GRP, gastrin-releasing peptide; GRPR, GRP receptor; hBRS3, human BRS3; mBRS3, mouse BRS3; NFAT, nuclear factor of activated T cells; NMB, neuromedin B; NMBR, NMB receptor; pERK, phosphorylated ERK; sBRS3, spotted gar BRS3; SRE, serum response element; SRF, serum response factor; tBRS3, turtle BRS3.

Since BRS3 of placental mammals underwent positive selection (Fig 1B), 17 vertebrate species (8 placental mammals: *Homo* and *Pan* represent Euarchonta, *Mus* and *Rattus* represent Glires, *Canis* and *Felis* represent Laurasiatheria, and *Orycteropus* and *Loxodonta* represent Atlantogenata; 3 nonplacental mammals: *Phascolarctos* and *Monodelphis* represent Marsupialia, and *Ornithorhynchus* represents Monotremata; 6 nonmammalian vertebrates: *Gallus* and *Corvus* represent bird, *Chrysemys* and *Anolis* represent reptile, *Xenopus* represents amphibian, and *Lepisosteus* represents fish), representing diverse types of vertebrates, were used for exploring potential structural changes underlying functional differences of BRS3 between placental mammals and nonplacental vertebrates. As shown in S4 Fig and S1 Fig, the average sequence identities and similarities of BRS3 ranged from 0.45 to 0.5 and 0.62 to 0.65 when each of the BRS3 receptors was compared to NMBRs/GRPRs. There was no significant difference between placental mammalian and nonplacental vertebrate BRS3 receptors (*P* = 0.164) when their amino acid sequences were compared to those of GRPR and NMBR on average (S4 Fig and S1A Fig). This result indicated that phylogenetic relationships did not reflect the pharmacological differences of BRS3 receptors [16]. Instead, examining the GPCR structures could provide important insights into their pharmacological features [1, 16]. Consequently, we predicted the 17 BRS3 structures using the Iterative Threading Assembly Refinement (I-TASSER) web server [27] (S1B Fig). Each of 17 BRS3 structures was compared with each structure of the NMBRs and GRPRs, respectively (S1B and S1C Fig). Two representative structures were shown in Fig 2 (human represents placental mammals, and turtle represents nonplacental vertebrates), and the other eight species were shown in S4 Fig. As shown in S1A Fig and S4 Fig, the average structural similarities between nonplacental vertebrate BRS3 and NMBR/GRPR were high with root-mean-square deviations (RMSDs) and ranged from 9.22 Å to 10.77 Å. In contrast, the placental mammalian BRS3 receptors had significantly (*P* ≈ 0.00 << 0.01) higher RMSDs (ranging from 13.82 Å to 14.38Å) compared to those of nonplacental vertebrate BRS3 (S1A Fig and S4 Fig). In contrast, all 17 vertebrate NMBRs showed no significant difference in structural similarities, with RMSDs ranging from 5.55 Å to 7.70 Å and 4.65 Å to 7.93 Å when compared to each of GRPRs, respectively (S2 Fig). Also, there is no significant difference when placental mammalian and nonplacental vertebrate NMBR structures were compared with each structure of GRPRs (S2 Fig). Furthermore, as shown in Fig 2, S1 Fig, and S4 Fig, there is a significant difference between the predicted N-terminal structures of placental mammalian BRS3 versus nonplacental vertebrate BRS3. The N-terminal structures of placental mammalian BRS3 consisted of a fragment of alpha helix and coils (Fig 2, S1 Fig and S4 Fig). In contrast, N termini of nonplacental vertebrate BRS3 show the coils structure, which is same with GRPR and NMBR in all vertebrates (Fig 2, S1 Fig, and S4 Fig). Taken together, our results indicated that after positive selection, the protein structure of placental mammalian BRS3 differed in comparison to nonplacental vertebrate BRS3, especially with respect to the N termini, possibly resulting in different pharmacological properties.

To verify the functional differences of BRS3 between placental mammals and nonplacental vertebrates, 10 vertebrate species (4 placental mammals: human/*Homo* represents Euarchonta, mouse/*Mus* represents Glires, dog/*Canis* represents Laurasiatheria, and aardvark/*Orycteropus* represents Atlantogenata; 2 nonplacental mammals: koala/*Phascolarctos* represents Marsupialia, and platypus/*Ornithorhynchus* represents Monotremata; 4 nonmammalian vertebrates: chicken/*Gallus* represents bird, turtle/*Chrysemys* represents reptile, frog/*Xenopus* represents amphibian, and spotted gar/*Lepisosteus* represents fish) were selected to test the biological experimental research (Fig 2 and S4 Fig). Meanwhile, since ERK phosphorylation and mobilization of calcium ions are common indicators of GPCR activation [28], stimulation of intracellular calcium and ERK by bombesin receptors requires ligand binding to stimulate Gq signaling [1, 11]. Two conserved consensus peptides, representing conserved mature peptides of GRP and NMB in vertebrates, were synthesized for receptor–ligand function assays [1, 11] (S3 Fig). These GRP and NMB peptides were utilized to activate the empty vector plasmid pcDNA3.1-V5-His (negative control group) and each of 10 BRS3 receptors, respectively. All 6 BRS3 receptors in nonplacental vertebrates (koala, platypus, chicken, turtle, frog, and spotted gar) could be activated by GRP and NMB, BRS3 receptors stimulated an increase in the levels of phosphorylated ERK (pERK) (Fig 2 and S4 Fig), and pERK levels were similar to those when GRPR was stimulated by GRP or NMBR stimulated by NMB (S4 Fig bottom). In contrast, all 4 BRS3 receptors in placental mammals (human, mouse, dog, and aardvark) could not be activated by GRP or NMB for ERK phosphorylation increase (Fig 2 and S4 Fig). Furthermore, there was a remarkable increase of Ca^2+^ ions in cells only when all 6 BRS3 receptors of nonplacental vertebrates were stimulated with GRP or NMB (Fig 2 and S4 Fig), and Ca^2+^ ion levels were similar to those when GRPR was stimulated by GRP or NMBR was stimulated by NMB (S4 Fig bottom). In contrast, no stimulation was observed in all four placental mammalian BRS3 (Fig 2 and S4 Fig). Also, we quantitated the ERK and calcium assays by statistical analysis in S5 Fig. Moreover, when we tested them in luciferase reporters, they showed results similar to the nuclear factor of activated T cells response element (NFAT-RE) luciferase reporter (S6B Fig) [29].

On the other hand, it is well known that many GPCRs are coupled to multiple G proteins, which lead to regulation of respective downstream signaling pathways [1]. The existence of ligand-independent (i.e., constitutive) activity of GPCRs was first described in the 1980s, and numerous additional constitutively active GPCRs have been reported to this day [17]. BRS3 was reported to be a Gq-coupled GPCR receptor in vertebrate systems [15]. Since BRS3 of placental mammals lost the connection with GRP and NMB, we investigated its potential constitutive activity with G protein luciferase functional assays, and cAMP response element (CRE), NFAT-RE, serum response factor response element (SRF-RE), and serum response element (SRE) luciferase reporters were utilized for testing Gs, Gq, G12, and potential Gi signaling in human embryonic kidney 293 (HEK293) cells, respectively [29]. In luciferase functional assays, BRS3 plasmids representing 10 classic species were cotransfected with four luciferase reporter plasmids, respectively. CRE, NFAT-RE, and SRF-RE luciferase units for placental mammalian BRS3 (human, mouse, dog, and aardvark) showed a significant increase in a dose-dependent manner (Fig 2, S4 Fig, and S6A Fig). Especially in the case of mouse BRS3 (mBRS3), the degree of activity increased with the increase of transfection concentration up to 19.0-fold, 18.6-fold, and 20.1-fold for CRE, NFAT-RE, and SRF-RE luciferase units, respectively (S4 and S5A Figs). Moreover, with a GTPgamma35S incorporation assay [30–32], we further confirmed that placental mammalian BRS3 coupled with Gs, Gq, and G12 and that nonplacental BRS3 coupled with Gq (Fig 2A and Fig 2B). Our data show that BRS3 expression in mouse brain tissue is even higher than that in mBRS3 highest dose–transfected cells (S7B Fig). In contrast, stimulation of luciferase units in a receptor in a dose-dependent manner could not be detected for BRS3 in all six nonplacental vertebrates (koala, platypus, chicken, turtle, frog, and spotted gar), including two nonplacental mammals (koala and platypus) (Fig 2, S4 Fig, and S5A Fig). Also, since Gs signaling mediates the cAMP stimulation, we tested the intracellular cAMP level, and the result was similar with luciferase reporter assay (S8 Fig). Moreover, when four luciferase reporters were cotransfected with BRS3 receptors of placental mammals, respectively, stimulation of luciferase units cannot be detected in a potential ligand (GRP/NMB) in a dose-dependent manner (S6B Fig). Taken together, our results revealed that nonplacental vertebrates and placental mammals show a different structure and different function probably because of adaptive evolution in placental mammals. Our results showed NMB and GRP are the endogenous cognate ligands for BRS3 in nonplacental vertebrates, whereas BRS3 of placental mammals constitutively activates Gs, Gq, and G12 signaling.

### GRP and NMB directly bind to BRS3 in nonplacental vertebrates with high affinity but not in placental mammals

**Fig 3:**
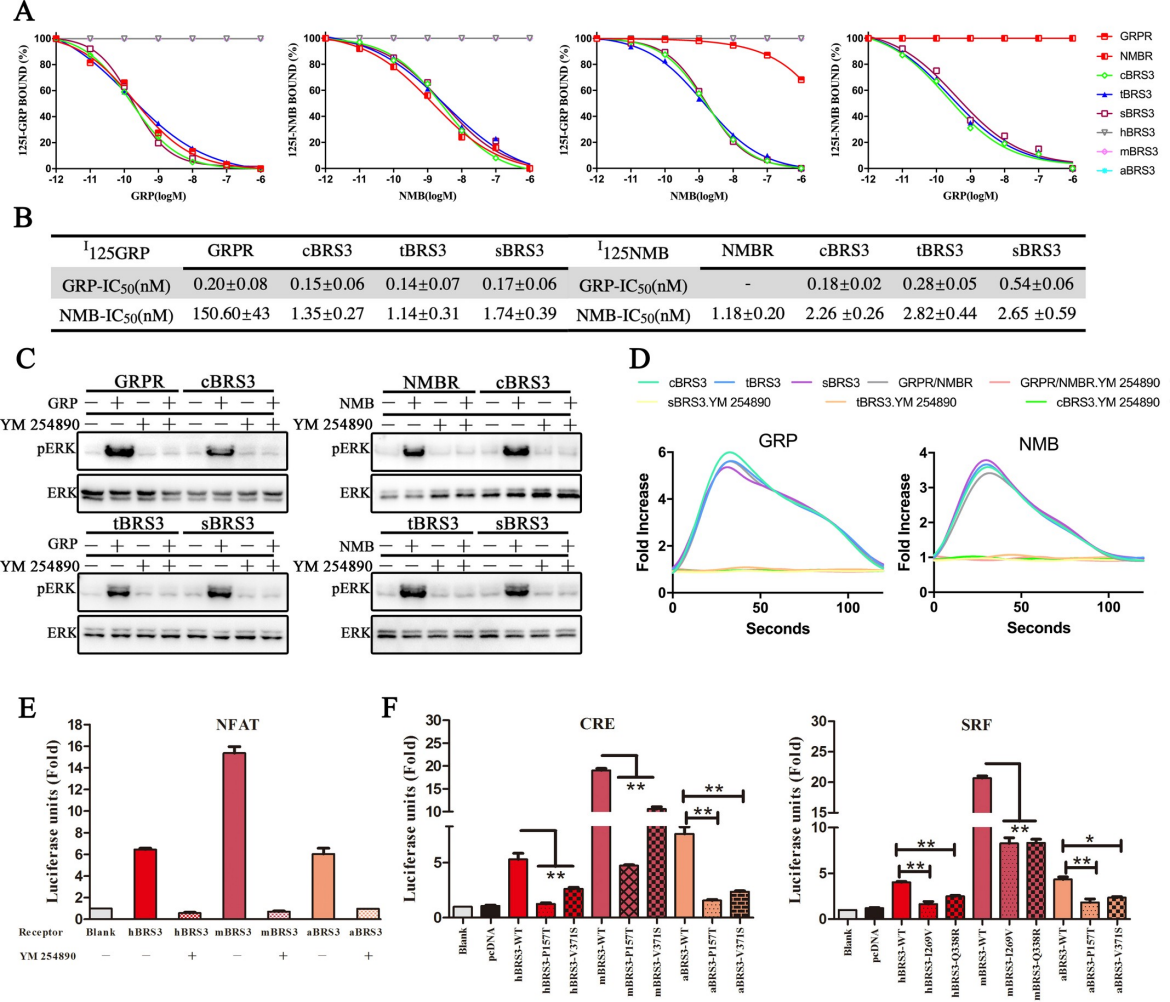
GRP and NMB directly bind to BRS3 in nonplacental vertebrates in high affinity via Gq signaling, whereas BRS3 in placental mammals constitutively activates two novel G protein–signaling Gs and G12. (A) Binding of GRP and NMB to BRS3 receptors in nonplacental vertebrates. (B) Comparison of binding affinity of GRP/NMB to BRS3, GRPR, and NMBR receptors. “-” indicates not detected. Values represent IC_50_ expressing as mean ± SE for at least three independent experiments. (C) YM 254890 inhibits the phosphorylation levels of ERK for BRS3 receptors in nonplacental vertebrates. Receptor-expressing HEK293 cells were pretreated with inhibitor for 1 h with 25 μM. Subsequently, GRP or NMB peptides were added to the cells at a concentration of 1 μM for 5 min before western blot. (D) YM 254890 inhibits Ca^2+^ ion levels in cells transfected with BRS3 receptors of nonplacental vertebrates. Receptor-expressing HEK293 cells were pretreated with inhibitor for 1 h with 25 μM. Subsequently, GRP or NMB were added to the cells at a concentration of 10 nM prior to the assay. The calcium fold is calculated by fluorescence intensity (excitation/emission wavelength: 490/520 nm). (E) YM 254890 inhibits the constitutively activated Gq signaling pathway with BRS3 of placental mammals. Receptor-expressing HEK293 cells were pretreated with 25 μM inhibitor for 12 h prior to the luciferase assays. (F) Constitutive activity for each BRS3 mutants in placental mammals. Statistical significance was defined as a *P* value < 0.05 (*) or *P* value < 0.01 (**). The underlying data can be found in S2 Data. aBRS3, aardvark BRS3; BRS3, bombesin receptor subtype-3; cBRS3, chicken BRS3; CRE, cAMP response element; ERK, extracellular signal–regulated kinase; GRP, gastrin-releasing peptide; GRPR, GRP receptor; hBRS3, human BRS33; HEK293, human embryonic kidney 293; mBRS3, mouse BRS3; NFAT, nuclear factor of activated T cells; NMB, neuromedin B; NMBR, NMB receptor; pERK, phosphorylated ERK; sBRS3, spotted gar BRS3; SRF, serum response factor; tBRS3, turtle BRS3.

To further verify our hypothesis, three classic BRS3 receptors in nonplacental vertebrates (chicken BRS3 [cBRS3], turtle BRS3 [tBRS3], and spotted gar BRS3 [sBRS3]) and three classic placental mammalian BRS3 receptors (human BRS3 [hBRS3], mBRS3, and aardvark BRS3 [aBRS3]) were selected for binding experimental research. As shown in Fig 3A and 3B, binding experiments also showed the direct binding of GRP to BRS3 in nonplacental vertebrates (cBRS3, tBRS3, and sBRS3) with high affinity (IC_50_ = 0.15 nM for cBRS3, IC_50_ = 0.14 nM for tBRS3, and IC_50_ = 0.17 nM for sBRS3), but no binding was detected in placental mammals (hBRS3, mBRS3, and aBRS3). Furthermore, we showed direct binding of NMB to BRS3 in nonplacental vertebrates (cBRS3, tBRS3, and sBRS3) with high affinity (IC_50_ = 2.26 nM for cBRS3, IC_50_ = 2.82 nM for tBRS3, and IC_50_ = 2.65 nM for sBRS3) but again no binding to placental mammalian BRS3 (hBRS3, mBRS3, and aBRS3) (Fig 3A and 3B). The binding affinities for GRP and NMB to BRS3 in nonplacental vertebrates were consistent with the GRP–GRPR and NMB–NMBR pairs (Fig 3A and 3B) [33]. We also confirmed that NMB inhibits GRPR–GRP binding with very low affinity and that GRP cannot inhibit NMBR–NMB binding (Fig 3A, Fig 3B, and S9 Fig) [33]. High affinity was observed when NMB inhibits BRS3–GRP binding in nonplacental vertebrates (IC_50_ = 1.35 nM for cBRS3, IC_50_ = 1.14 nM for tBRS3, and IC_50_ = 1.74 nM for sBRS3) and when GRP inhibits BRS3–NMB binding in nonplacental vertebrates (IC_50_ = 0.18 nM for cBRS3, IC_50_ = 0.28 nM for tBRS3, and IC_50_ = 0.54 nM for sBRS3) (Fig 3A and 3B). In summary, our results showed that BRS3 in nonplacental vertebrates can still bind to the original ligands, NMB and GRP, with high binding affinity and be activated to stimulate Gq signaling (Fig 2, Fig 3A and 3B, and S4 Fig). The BRS3s in placental mammals have apparently lost this activity because of their inability to bind those ligands (Ki > 1,000 nM).

### BRS3 in placental mammals constitutively activates two novel GPCR signaling pathways—Gs and G12—because of positive selection

We further verify that BRS3 in placental mammals constitutively activates Gs, Gq, and G12 signaling. Since Gs and G12 signaling show independent signaling pathways but Gi and Gq signaling showed some similar downstream activation (e.g., ERK phosphorylation), a specific Gq signaling inhibitor YM 254890 was used to confirm Gq signaling of BRS3 receptors [34–36]. We obtained additional confirmation that ERK phosphorylation and calcium ion mobilization were inhibited in the case of BRS3 in three nonplacental vertebrates (cBRS3, tBRS3, and sBRS3) and GRP/NMB peptide ligands (Fig 3C and 3D). Furthermore, YM 254890 inhibition confirmed the constitutive activity of BRS3 receptors in three placental mammals (hBRS3, mBRS3, and aBRS3) via Gq signaling (Fig 3E). Also, when we apply another Gq downstream signaling inhibitor, sotrastaurin, to these experiments, similar results are shown (S10 Fig).

BRS3s in both placental mammals and nonplacental vertebrates were coupled with Gq signaling (Fig 3), but only BRS3 of placental mammals was coupled with Gs and G12 signaling (Fig 2 and S4 Fig). The Gs and G12 selectivity barcodes were recognized by different key residues of GPCR to trigger a specific downstream pathway [1]. The largest possible binding pocket of Gs, G12, and GPCR was predicted by Discovery Studio 3.0 [37]. These pockets were utilized to construct an initial coarse model of the protein–protein complex, and the model with the lowest energy was then obtained using RosettaDock [38, 39]. Then, binding sites were obtained by the Residue Interaction Network Generator (RING) [40], and the results were shown in S2 Table. Thus, four amino acids (157P, 371V, 269I, and 338Q) were predicted as key residues of placental mammalian BRS3 to trigger a specific downstream pathway, since they exhibited a large degree of conservation in mammals, and they differ from the corresponding amino acids in nonplacental vertebrate species (S11 and S12 Figs). As shown in Fig 3F, these placental mammalian amino acids change back to the nonplacental vertebrate (turtle/chicken) residues and trigger Gs and G12 signaling, as manifested in a decreased stimulation in the CRE and SRF-RE luciferase assay. GPCR expression patterns in cells were examined after plasmid transfection (S7 Fig). The residue 338Q is under positive selection (Fig 1B), and the other three residues (157P, 371V, and 269I) are consistently changed and conserved in placental mammals (S11 and S12 Figs). We also tested eight neighboring residues to check specificity of the amino acids we predicted, and the neighboring residues have no effect or little effect except for reduced expression on the receptor activity (S13 Fig). Taken together, our results revealed that BRS3 in placental mammals constitutively activates two novel GPCR signaling pathways—Gs and G12—because of positive selection.

### Key binding/activating sites of BRS3 in nonplacental vertebrates for recognition of GRP and NMB are altered to key residues that regulate constitutive activity in placental mammalian BRS3

**Fig 4:**
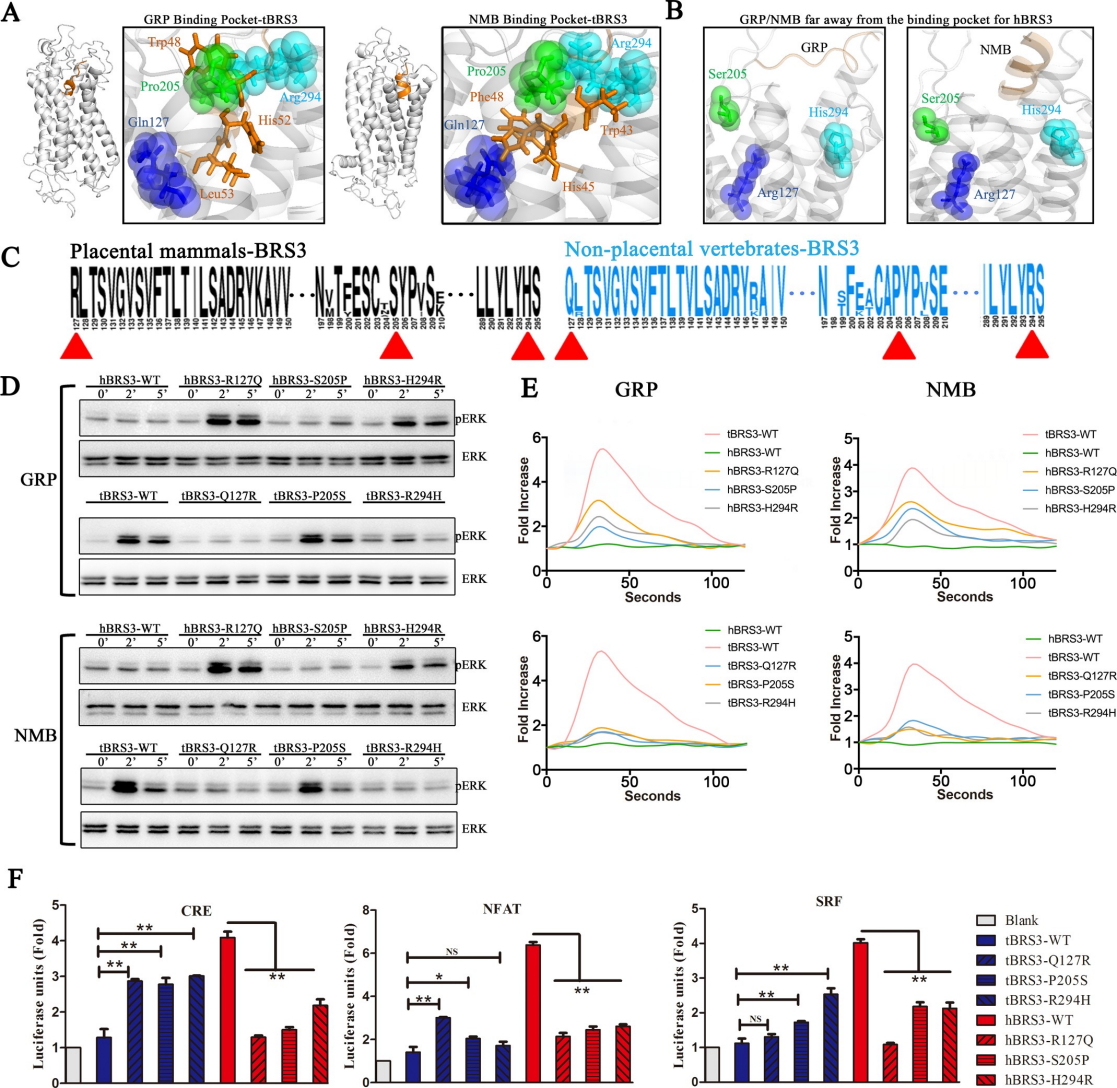
Key interaction sites of BRS3 in nonplacental vertebrates with GRP or NMB peptides regulate constitutive activity of placental mammalian BRS3 (human versus turtle). (A) Potential key interaction sites of tBRS3 with GRP or NMB peptides. Spheres and sticks represent sites of BRS3 and sites of GRP/NMB, respectively. (B) GRP/NMB is further away from the binding pocket of hBRS3. (C) Sequence logo of BRS3 receptors in placental mammals and nonplacental vertebrates. Red triangles represent positions of potential key interaction sites of BRS3. (D) The phosphorylation levels of ERK for each of three tBRS3 mutants and three hBRS3 mutants. (E) The level of Ca^2+^ ions in cells for each of three tBRS3 mutants and three hBRS3 mutants. The calcium fold is calculated by fluorescence intensity (excitation/emission wavelength: 490/520 nm). (F) Constitutive activity for each of three tBRS3 mutants and three hBRS3 mutants. Statistical significance was defined as a *P* value < 0.05 (*) or *P* value < 0.01 (**). The underlying data can be found in S3 Data. BRS3, bombesin receptor subtype-3; CRE, cAMP response element; ERK, extracellular signal–regulated kinase; GRP, gastrin-releasing peptide; hBRS3, human BRS3; NFAT, nuclear factor of activated T cells; NMB, neuromedin B; NS, not significant; pERK, phosphorylated ERK; SRF, serum response factor; tBRS3, turtle BRS3; WT, wild type.

**Fig 5:**
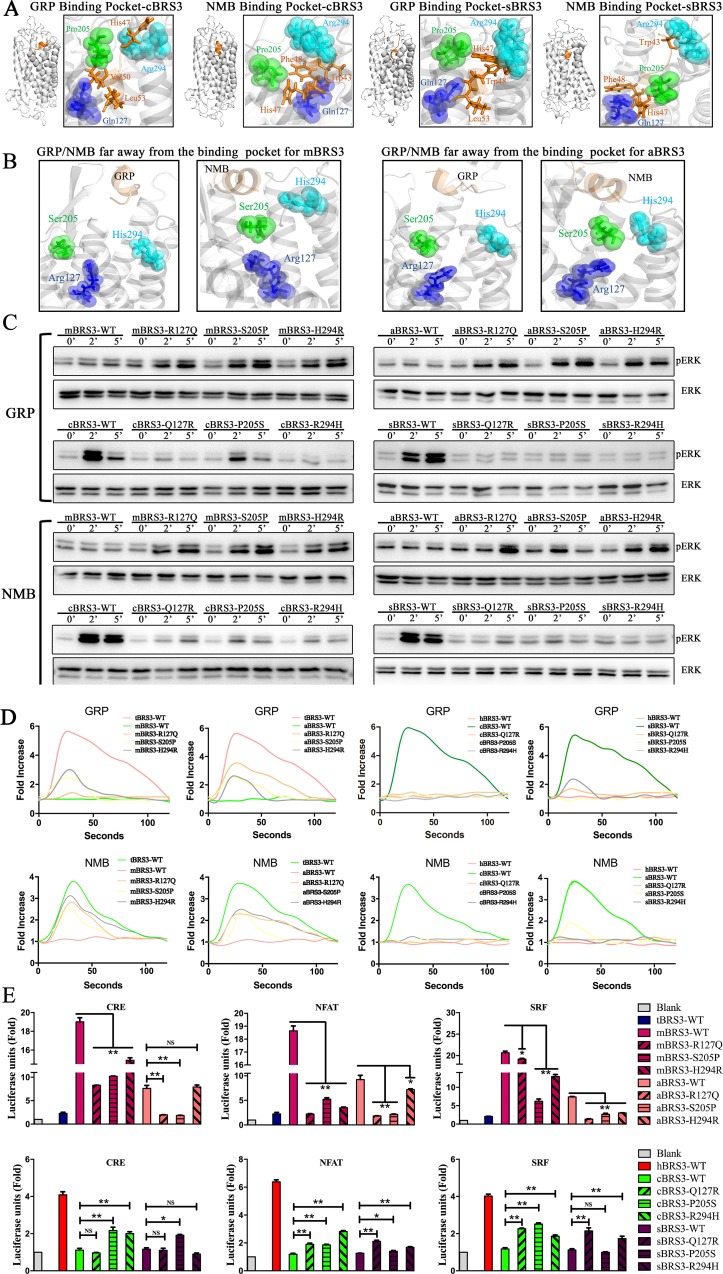
Key interaction sites of BRS3 in nonplacental vertebrates with GRP or NMB peptides regulate constitutive activity of placental mammalian BRS3 (mouse/aardvark versus chicken/spotted gar). (A) Potential key interaction sites of BRS3 in nonplacental vertebrates (cBRS3, sBRS3) with GRP or NMB peptides. Spheres and sticks represent sites of BRS3 and sites of GRP/NMB. (B) GRP/NMB is further away from the binding pocket of BRS3 in placental mammals (mBRS3, aBRS3). (C) The phosphorylation levels of ERK for each of three BRS3 mutants (in mBRS3, aBRS3, cBRS3, sBRS3). (D) The level of Ca^2+^ ions in cells for each of three BRS3 mutants (in mBRS3, aBRS3, cBRS3, sBRS3). The calcium fold is calculated by fluorescence intensity (excitation/emission wavelength: 490/520 nm). (E) Constitutive activity for each of three BRS3 mutants (in mBRS3, aBRS3, cBRS3, sBRS3). Statistical significance was defined as a *P* value < 0.05 (*) or *P* value < 0.01 (**). The underlying data can be found in S4 Data. aBRS3, aardvark BRS33; BRS3, bombesin receptor subtype-3; cBRS3, chicken BRS3; CRE, cAMP response element; ERK, extracellular signal–regulated kinase; GRP, gastrin-releasing peptide; mBRS3, mouse BRS3; NFAT, nuclear factor of activated T cells; NMB, neuromedin 3; NS, not significant; pERK, phosphorylated ERK; sBRS3, spotted gar BRS3; SRF, serum response factor; WT, wild type.

In order to explore the key binding/activating sites of BRS3 with its endogenous ligands, GRP and NMB, in nonplacental vertebrates, receptor–ligand docking was completed by a combination of the Discover Studio package and the FelxPepDock module of Rosetta [41]. Discovery Studio 3.0 was utilized for predicting the potential binding pockets of BRS3 receptors, the largest possible binding pocket was utilized to construct an initial coarse model of the peptide-protein complex, and the initial model was obtained using RosettaDock [37]. Since the peptide was considered as a rigid body in RosettaDock, the peptide–protein complex with the lowest energy was then refined by utilizing the FelxPepDock module of Rosetta. The output of 2,000 models was then ranked based on their energy score. The first 10 low-energy-score models for each of the three types of receptor–ligand complexes were selected to further analyze the binding sites. Three residues of nonplacental vertebrate BRS3—namely, 127Q, 205P, and 294R—were frequently found to be the binding sites for each of nonplacental vertebrate receptor–ligand complexes (BRS3–GRP and BRS3–NMB) (Figs 4A and 5A and S3 Table). In contrast, the three corresponding residues in placental mammalian BRS3 (127R, 205S, and 294H) were found to have moved away from the ligands (Figs 4B and 5B). Furthermore, according to sequence logos between nonplacental vertebrate BRS3 and placental mammalian BRS3, the three residues were conserved in nonplacental vertebrate BRS3 and altered in placental mammalian BRS3 sequences (Fig 4C, S11 Fig and S12 Fig). Therefore, combined with other results previously reported [42–44], our results indicated the three residues 127Q, 205P, and 294R as the potential key binding sites of nonplacental vertebrate BRS3 for binding GRP and NMB peptides.

To further investigate the function of the three residues (127Q, 205P, and 294R) in the receptor–ligand pocket, the three residues in BRS3 of three classic nonplacental vertebrates (cBRS3, tBRS3, and sBRS3) were mutated to the corresponding residues in the placental mammalian orthologs, respectively. In contrast, the three residues (127R, 205S, and 294H) in placental mammalian BRS3 (hBRS3, mBRS3, and aBRS3) were reversely mutated to the corresponding residues in nonplacental vertebrate BRS3, respectively. The mutants (Q127R, P205S, and R294H) for cBRS3, tBRS3, and sBRS3 and the reverse mutants (R127Q, S205P, and H294R) for hBRS3, mBRS3, and aBRS3 were constructed to further confirm the important receptor–ligand binding/activating sites of BRS3. GPCR expression patterns in cells were examined after plasmid transfection (S7A Fig). As shown in Fig 4D, GRP and NMB stimulation of ERK phosphorylation almost could not be detected in the Q127R and R294H mutants in the tBRS3 background; except for the P205S mutant, the effect is not obvious. Simultaneously, the GRP/NMB-induced ERK phosphorylation signal could be detected in the R127Q and H294R mutants, and the effect is not obvious in the S205P mutant for the corresponding hBRS3 background (Fig 4D). Furthermore, as shown in Fig 4E, there was a remarkable decrease of cellular Ca^2+^ ions for each of three mutants (Q127R, P205S, and R294H) of tBRS3 receptor compared with that of wild-type tBRS3, when GRP and NMB were utilized to stimulate (Fig 4E). In contrast, a remarkable increase of cellular Ca^2+^ ions for each of three mutants (R127Q, S205P, and H294R) in the hBRS3 receptor background was found when compared to Ca^2+^ levels in wild-type hBRS3, when GRP and NMB were utilized to stimulate each of the three mutants (Fig 4E). Similar results were obtained when we tested the R127Q, S205P, and H294R mutants in the genes of other placental mammalian species (mBRS3 and aBRS3) and the Q127R, P205S, and R294H mutations in the corresponding genes of nonplacental vertebrates (cBRS3 and sBRS3) (Fig 5C). In contrast, all three mutants, including S205P in mBRS3 and aBRS3, lead to ERK phosphorylation signal and a significant increase in cellular Ca^2+^ ions levels (Fig 5D). Also, the significant effects for both ERK phosphorylation and cellular Ca^2+^ ions levels can be detected in three mutants (only excepting P205S of cBRS3 for ERK phosphorylation activated by NMB) of cBRS3 and sBRS3 (Fig 5C and 5D). When we apply these to luciferase reporters, results were similar to those in ERK and Ca^2+^ assays (S14 Fig). These results indicated that each of the three residues (i.e., 127Q, 205P, and 294R) might play a critical role in the process of BRS3 with GRP/NMB interactions and that the 205th residue differs in function in different species.

The impact of point mutations for each of the three residues with respect to placental mammalian BRS3 constitutive activity was also investigated. As shown in Fig 4F, CRE, NFAT-RE, and SRF-RE luciferase units for each of R127Q, S205P, and H294R mutants of hBRS3 were significantly decreased in comparison to those of wild-type hBRS3. Especially, the mutant R127Q leads to the most significant reduction of the three signal pathways (Fig 4F). The three mutants of tBRS3 were also tested. In contrast, almost three mutants (Q127R, P205S, and R294H) of tBRS3 had a significant increase when compared to those of wild-type tBRS3 (Fig 4F). Simultaneously, the three corresponding mutants of the other two placental mammals (mBRS3 and aBRS3) and the two nonplacental vertebrates (cBRS3 and sBRS3) were tested, and the results were consistent with the aforementioned hBRS3 and tBRS3 mutants, respectively (Fig 5E).

Our results revealed that 127Q, 205P, and 294R were the key residues for BRS3 in nonplacental vertebrate recognition of GRP or NMB peptides (Figs 4A and 5A). As for the three key residues, 127R and 294H are under positive selection (Fig 1B), and 205S is consistently changed and conserved in placental mammals (Fig 4C). Taken together, our results suggested that positive selection in placental mammalian BRS3 leads to the disconnection between BRS3 and its original ligands, GRP/NMB. Furthermore, each of the three residues (i.e., 127R, 205S, and 294H) had an impact on the process of placental mammalian BRS3 constitutive activation via the attached G protein, resulting in activation of three signal transduction pathways (Gs, Gq, and G12). However, when we mutated all three residues (R127Q, S205P, and H294R), the results of triple mutants were similar to those of single mutants, and we cannot interpret it appropriately (S15 Fig). Therefore, the key binding/activating sites of BRS3 in nonplacental vertebrates for recognition of GRP and NMB are altered to key residues that regulate constitutive activity in placental mammalian BRS3. Our results suggested placental mammalian BRS3 underwent positive selection to lose the connection to their original peptide ligands and eventually to evolve into a constitutively active GPCR.

### N terminus undergoes positive selection, resulting in constitutive activity of placental mammalian BRS3

**Fig 6:**
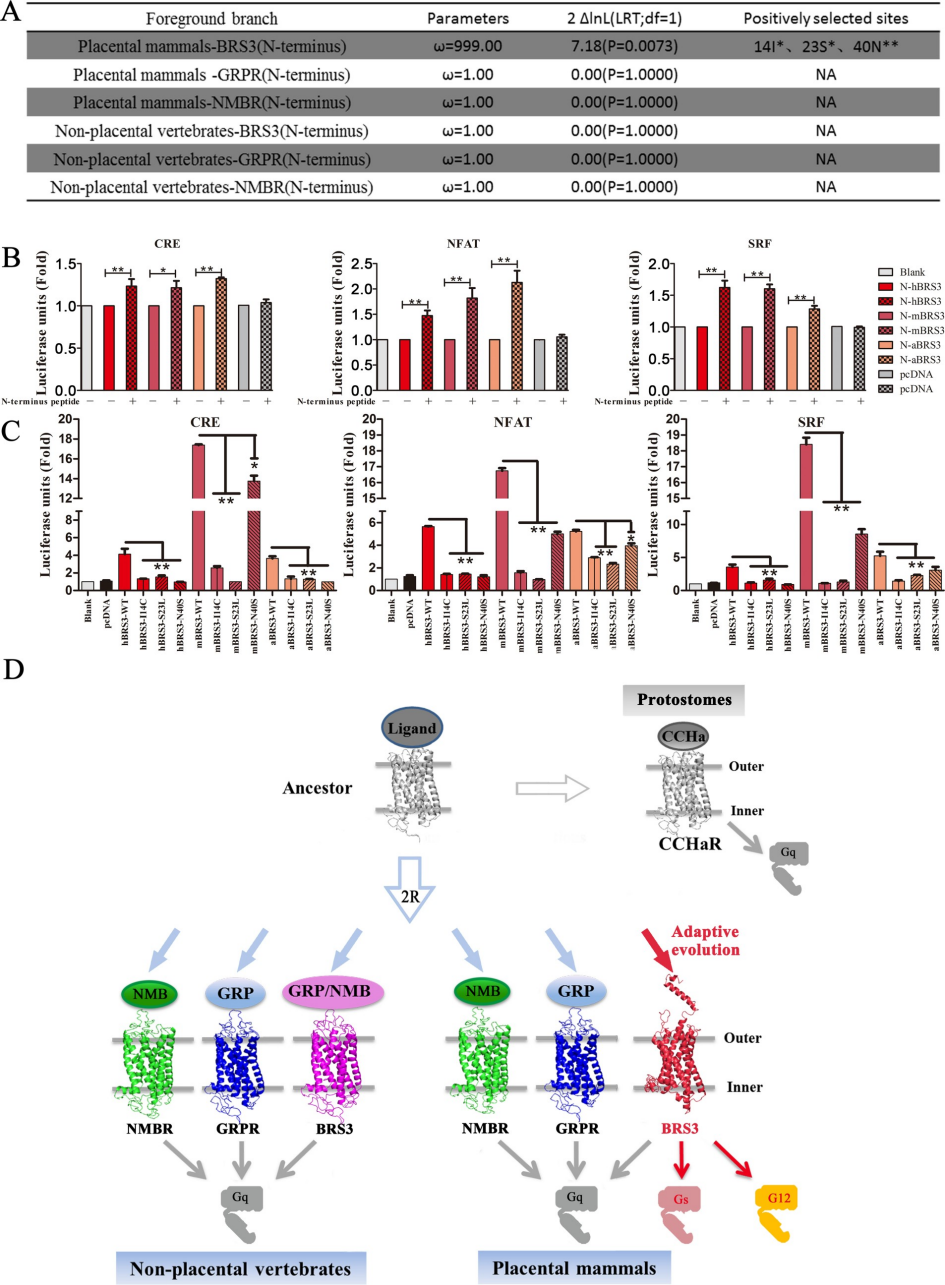
The N terminus of BRS3 in placental mammals underwent positive selection for its constitutive activity. (A) The positively selected sites for the N terminus of BRS3, GRPR, and NMBR in placental mammals and nonplacental vertebrates; ** and * indicate *P* values exceeding 0.99 and 0.95, respectively. (B) N-BRS3 receptors of placental mammals can be stimulated by N-terminal peptide of mBRS3; * indicates *P* < 0.05; ** indicates *P* < 0.01. (C) Constitutive activity for positively selected BRS3 mutants in placental mammals. (D) Diagram of evolution of BRS3 receptor and the signaling pathway conversion. The underlying data can be found in S5 Data. 2R, two rounds of whole-genome duplications; aBRS3, aardvark BRS3; BRS3, bombesin receptor subtype-3; CCHaR, CCHamide receptor; CRE, cAMP response element; GRP, gastrin-releasing peptide; GRPR, GRP receptor; hBRS3, human BRS3; LRT, likelihood ratio test; mBRS3, mouse BRS3; NA, not available; NFAT, nuclear factor of activated T cells; NMB, neuromedin B; NMBR, NMB receptor; SRF, serum response factor.

To explore the role of the N terminus in the placental mammalian BRS3, the phylogenetic analysis by maximum likelihood (PAML) method was applied to test for positive selection of the N terminus (41 amino acids), which consists of a fragment of alpha helix and coils, and showed a significant difference between placental mammals and nonplacental vertebrates (Fig 2 and S4 Fig) [45]. Truncated receptor expression patterns in cells were examined after plasmid transfection (S7A Fig). The N-terminal domain of placental mammalian BRS3 exhibit a large nonsynonymous (dN)/synonymous (dS) substitution rate ratio (branch-site dN/dS of ω >> 1) that is highly significant (LRT, *P* < 0.05; Fig 6A) but not in nonplacental vertebrate orthologs. Three residues (14I, 23S, and 40N) were found to have undergone positive selection (Fig 6A). Constitutive activation of mBRS3 is the most significant compared with the other two placental mammalian species (S4 Fig); therefore, a synthetic N terminus peptide of mBRS3 and luciferase functional assays for placental mammalian BRS3 with truncated N termini (N-hBRS3, N-mBRS3, and N-aBRS3) were investigated to further verify the role of this domain in for constitutive activity. When these N-BRS3 receptors were stimulated by an N-terminal peptide of mBRS3, all CRE/NFAT-RE/SRF-RE signaling pathways could be activated (Fig 6B). In contrast, N-cBRS3, N-sBRS3, and N-tBRS3 receptors cannot be stimulated by an N-terminal peptide, unlike placental mammalian BRS3 (S16 Fig). The stimulation by the N-terminal peptide is not as high as the one achieved with the entire wild-type hBRS3, presumably because the synthetic exogenous peptide does not form the necessary structures with the remainder of the receptor for optimal functionality [46]. Besides, we test the truncated BRS3 receptors’ constitutive activation level with luciferase reporter in HEK293 cells. The result showed that truncated receptors also have significant but lower stimulations, compared with intact BRS3 (S17 Fig).

These positive selection sites (14I, 23S, and 40N) were conserved amino acids in placental mammalian BRS3 (S11 and S12 Figs). Therefore, the three residues were considered as key residues for constitutive activity of BRS3 in placental mammals. To test these key residues, we mutated these residues in hBRS3, mBRS3, and aBRS3 to residues found in the nonplacental vertebrate (turtle/chicken 14C, 23L, and 40S). After transfection in cultured HEK293 cells, luciferase functional assays were utilized to test signaling. As shown in Fig 6C, all mutants of both hBRS3 and aBRS3 exhibited a significant decrease compared with wild-type hBRS3 and aBRS3, respectively. Especially when S23L was tested for the mouse gene, all CRE/NFAT-RE/SRF-RE luciferase units were sharply reduced to background levels (Fig 6C). To further exam the positively selected sites in the N terminus of BRS3, we also predicted N-terminal triple-mutated structures of BRS3, and these mutations do modify the secondary structure of the N terminus of BRS3 to non-alpha helix (S18 Fig). These results revealed that the three N-terminal residues (14I, 23S, and 40N) were key residues for the constitutive activity of placental mammalian BRS3. Taken together, our results suggested that the N terminus plays a critical role in the conversion of the ancestral BRS3 receptor to constitutive activity in placental mammals.

## Discussion

BRS3 belongs to a classic polypeptide family, originated from the 2R event during vertebrate evolution sharing a common ancestry with NMBR and GRPR (Fig 1A). The latter two GPCRs and their peptide ligands coevolved and are conserved in vertebrates. It is well known that GPCRs connected with peptide ligands over the entire range of vertebrate evolution. Therefore, BRS3 was always considered as a classic orphan receptor since 1980s [7, 24]. Many synthetic agonists and antagonists are designed for pairing with BRS3, but up to now, no endogenous BLP as a cognate ligand of BRS3 has been identified [14, 15]. In this study, we showed that after the common ancestor of NMBR/GRPR/BRS3 expanded to three GPCRs in vertebrates, while NMBR paired with its endogenous peptide (NMB) in vertebrates and GRPR paired with its endogenous peptide (GRP) in vertebrates, BRS3 in nonplacental vertebrates is still connected to GRP and NMB with high binding affinity stimulating Gq signaling (Figs 1–3). In contrast, placental mammalian BRS3 lost the original connection with GRP and NMB (Fig 2, Fig 3 and S4 Fig). Moreover, positive selection of the BRS3 occurred in the placental mammalian lineage, and certain structures changed significantly compared with BRS3 of nonplacental vertebrates (Fig 1B, Fig 2 and S4 Fig). Under positive selection, with three altered key residues (R127Q, S205P, and H294R) affecting ligand binding and activation, placental mammalian BRS3 lost connection with its original ligands and became constitutively regulated by its altered G protein selectivity barcodes and its altered N terminus, which also underwent positive selection in placental mammals (Figs 3–6). Therefore, our results showed that the cognate ligand-BLPs for BRS3 actually are NMB and GRP, but only in nonplacental vertebrates, including nonplacental mammals (Marsupialia and Monotremata) (Fig 2, Fig 3 and S4 Fig), which is consistent with previous study [47]. In contrast, placental mammalian BRS3 underwent positive selection to become a constitutive active GPCR in a ligand-independent manner (Fig 2, Fig 3 and S4 Fig). Many studies have reported that the mutations from almost every part of GPCR can influence its constitutive activity [48, 49], and here our results consistently showed adaptive evolution of both original ligand-binding/activating sites and that the N-terminal domain drove the constitutive activity of placental mammalian BRS3 (Figs 5 and 6). In phylogenetic analysis and experiment data, platypus (representing Monotremata) and koala (representing Marsupialia) still harbor a nonplacental vertebrate-type BRS3 ortholog, but the aardvark (representing Atlantogenata) features a placental mammalian-type BRS3 ortholog (Fig 2, Fig 3 and S4 Fig). Also, evolution analysis for both whole gene and the N terminus of BRS3 found positive selection occurred after Marsupialia split from placental mammals (Figs 1 and 6A). Taken together, the endogenous BLP for placental mammalian BRS3 actually does not exist, and the constitutively active BRS3 is a genuinely orphan GPCR in placental mammals, including humans (Fig 6D).

At the organ level, NMBR and GRPR, members of the bombesin receptor family, are widely expressed, especially in the gastrointestinal tract and central nervous system (CNS), and have similar functions in regulating smooth muscle contraction and CNS effects [50]. In contrast, BRS3 expression appears to be highly species-dependent [51]. Most BRS3 acts in the brain, including regulating sympathetic outflow and affecting food intake, metabolic rate, body temperature, heart rate, blood pressure, and insulin secretion [50, 52, 53]. The best-established role of BRS3 is in the regulation of food intake, energy expenditure, and body weight [50, 53]. Consequently, BRS3 gene inactivation in mice causes obesity, whereas synthetic agonists produce weight loss [50, 53]. Here, we propose that placental mammalian BRS3 underwent positive selection in the placental mammal lineage to lose the connection with NMB/GRP and may carry out some physiological role. Further studies on BRS3 are necessary to unveil its different functions between nonplacental vertebrates and placental mammals (Fig 6D). However, one possibility that should not be ruled out is that after positive selection, placental mammalian BRS3 now binds a completely different endogenous ligand by chance. We cannot exclude this remote possibility, as it is virtually impossible to experimentally exclude all possible endogenous ligands for placental mammalian BRS3.

GPCR–ligand pairs coevolved and maintained a large degree of conservation concerning their physiological function and G protein signaling across vertebrates, with even more conservation in mammals [1, 28]. On the other hand, subtle differences of the same GPCR–ligand pairs were reported between human and mouse [54]. Our results also showed differences in signaling of the same point mutations generated in the mouse or human BRS3 gene (Figs 4 and 5). The difference was distributed not only in the stimulation of G protein signaling but also in key residues of the N terminus and other domains (Figs 4–6), which is consistent with significant pharmacological differences between rat and human that were reported previously [55]. Our results indicated diverse evolution between primates and rodents after origination of placental mammalian BRS3, and further studies are necessary to investigate the different function of BRS3 between human and mouse.

Coevolution of GPCR–ligand pairs appears more pronounced in the polypeptide family, and there are approximately 30 orphan GPCRs from the polypeptide family, in which endogenous peptide ligands are predicted to bind to and activate these orphan GPCRs [56]. Since GPCRs from the polypeptide family were considered to be paired with natural polypeptide ligands, many studies aim to identify endogenous peptides to deorphanize these orphan GPCRs in the polypeptide family [7, 16]. According to the evolution of GPCR-peptide pairs, after the 2R event during the origin of vertebrates, most orphan GPCRs arose via gene duplication and belong to specific subfamilies—e.g., GPR37L and GPR37 belong to the endothelin/cholecystokinin subfamily, BRS3 belongs to the NMB/GRP subfamily, and GPR39 belongs to the ghrelin/motilin subfamily [57, 58]. Although these orphan GPCRs were considered to be activated by a naturally occurring peptides, which should be similar to peptides in the same subfamily, they still have not been deorphanized during a long time period. Even though GPR39 has been reported to be activated by obestatin [59], a contrary view remains that GPR39 cannot be activated by obestatin, and it actually has been proposed to be a constitutively active GPCR in a ligand-independent manner [60, 61]. Similarly, GPR37 and GPR37L1 were deorphanized by neuropeptides head peptide [62] and prosaptide/prosaposin [63], but a number of questions remain regarding the pairing of these peptide ligands with GPR37 and GPR37L1 [64]. Thus far, GRP37 still is considered an orphan GPCR and has been reported to regulate cellular protein quality control during Wnt-signaling in a ligand-independent manner [64, 65]. GPR37 also exhibits N-terminal structural differences in comparison to its homologous GPCR, EDNRA/B, whereby the N terminus plays an important role in regulating its constitutive activity [66, 67]. Similarly, the N terminus of placental mammalian BRS3 and the N-terminal peptide of most adhesion GPCRs show a similar situation concerning constitutive activity (Fig 6) [46]. Taken together, those orphan GPCRs that cannot be deorphanized often showed constitutive activity. Thus, the contrary view that there exist endogenous ligands for these orphan GPCRs or that they only function constitutively in a ligand-independent manner has not been illuminated [21].

More than 140 orphan receptors that were considered to have endogenous ligands attracted a great deal of interests for deorphanization [3, 68]. However, the rate of GPCR deorphanization decreased drastically at the turn of this century, suggesting some gaps exist. The reason was mostly considered to be the lack of signal transduction assays and positive control experiments for these orphan GPCRs [69]. Some orphan GPCRs, which function in a ligand-independent manner, often cannot be paired with any of the possible endogenous ligands [19, 20, 60, 61, 66, 67]. Under these circumstances, we propose a new point of view that perhaps a large fraction of orphan GPCRs do not have endogenous ligands. Therefore, our results demonstrated that the BRS3 lost its endogenous ligand because of positive selection in placental mammals and finally functions constitutively to become a genuinely orphan GPCR in placental mammals, including humans. This should strengthen the view that at least some of the remaining orphan GPCRs will never be deorphanized by discovery of a natural ligand and will remain genuinely orphan GPCRs. Taken together, our study identified the first example that might represent a new group of GPCRs that will never be deorphanized by the discovery of a natural ligand and will remain genuinely orphan GPCRs that function constitutively in a ligand-independent manner, and it provided new perspectives in addition to the current ligand-driven GPCR deorphanization.

## Materials and methods

### Phylogenetic analysis and selection analysis of the bombesin receptor family

The exons of bombesin receptor family members and two paralogous genes (CCHaR-1 and CCHaR-2) and an out-group gene (EDNRA) were translated into the amino acid sequence and aligned with ClustalX version 1.8, using default settings [70]. The corresponding species and genes were shown in S1 Table. Unrooted tree topology based on multiple alignments of amino acid sequences was obtained using the Maximum Likelihood method in MEGA 6.06 [71].We used the branch-site model of PAML version 4.4 to test for positive selections on interested. The species for phylogenetic analysis were as follows: *Homo*: *H*. *sapiens*; *Pan*: *P*. *troglodytes*; *Mus*: *M*. *musculus*; *Rattus*: *R*. *norvegicus*; *Sus*: *S*. *scrofa*; *Capra*: *C*. *hircus*; *Ovis*: *O*. *aries*; *Canis*: *C*. *lupus familiaris*; *Felis*: *F*. *catus*; *Orycteropus*: *O*. *afer*; *Loxodonta*: *L*. *africana*; *Phascolarctos*: *P*. *cinereus*; *Monodelphis*: *M*. *domestica*; *Ornithorhynchus*: *O*. *anatinus*; *Gallus*: *G*. *gallus*; *Anolis*: *A*. *carolinensis*; *Chrysemys*: *C*. *picta*; *Xenopus*: *X*. *tropicalis*; *Lepisosteus*: *L*. *oculatus*; *Danio*: *D*. *rerio*; *Saccoglossus*: *S*. *kowalevskii*; *Acanthaster*: *A*. *planci*; *Strongylocentrotus*: *S*. *purpuratus*; *Apis*: *A*. *mellifera*; *Nasonia*: *N*. *vitripennis*; *Drosophila*: *D*. *melanogaster*; *Aedes*: *A*. *aegypti*; *Tribolium*: *T*. *castaneum*; *Camponotus*: *C*. *floridanus*; *Parasteatoda*: *P*. *tepidariorum*; *Myzus*: *M*. *persicae*; *Lingula*: *L*. *anatina*; *Crassostrea*: *C*. *virginica*; and *Mizuhopecten*: *M*. *yessoensis*.

### Genetic and structural analysis

WebLogo was developed to generate sequence logos that are graphical representations of the patterns within a multiple sequence alignment and to assist in discovering and analyzing those patterns [72, 73]. We implemented WebLogo to find conserved sites/areas in GPCRs. The sequence similarity and identity of GPCRs were computed by Sequence Manipulation Suite. The I-TASSER algorithm has been developed for protein conformation prediction [74, 75]. The structures of GPCRs were predicted using I-TASSER. The RMSD between structures of GPCRs was calculated by Rosetta software. Discovery Studio is a suite of packages for predicting the potential binding pockets of receptors. RosettaDock is a Monte Carlo–based multiscale docking algorithm that optimizes both rigid-body orientation and side-chain conformation [41]. According to the predicted largest possible binding pocket, an initial conformation of the protein complex was constructed for utilizing in RosettaDock. The FelxPepDock module of Rosetta is designed to create high-resolution models of complexes between flexible peptides and globular proteins [76]. Therefore, the peptide–protein complex with the lowest energy was then optimized by using the FelxPepDock module of Rosetta. The binding sites were analyzed based on the first 10 low-energy-score-optimized models of peptide–protein complexes. PyMOL is a molecular graphics system for the visualization of three-dimensional (3D) structures of GPCRs [77].

### Peptides and generation of BRS3 mutants

As for ligand peptides, two conserved and consensus peptides (GRP-GSHWAVGHLM-NH_**2**_ and NMB-GNLWATGHFM-NH_**2**_) and the N-terminal peptides of mBRS3 (MSQRQSQSPNQTLISITNDTETSSSVVSNDTTHKGWTGDNS-NH_**2**_) were synthesized from Biotech Bioengineering (Shanghai, China) for receptor–ligand function assays, except radioligand binding assays. BRS3 mutants were generated by introducing point mutations using a QuikChange II site-directed mutagenesis kit (Agilent Technologies). Briefly, overlapping primers with the desired point mutations were used to amplify wild-type BRS3. The parental plasmid was digested using the DpnI enzyme, and the newly synthesized plasmid was used as a template for PCR amplification of BRS3 mutants before subcloning into the pcDNA3.1-V5-His plasmid.

### Luciferase assays

To detect constitutive activity of BRS3, HEK293 cells were seeded in 24-well plates and cotransfected with the CRE/NFAT-RE/SRF-RE/SRE luciferase reporter plasmids (50 ng), the BRS3 from different species, or BRS3 mutants with different doses (10 ng/50 ng/150 ng); after 24 h, cells were further incubated in serum-free media for another 12 h before luciferase assay. As for peptide supplementation treatment, HEK293 cells were cotransfected with the CRE/NFAT-RE/SRF-RE/SRE luciferase reporter plasmids (50 ng) and various genes (300 ng) of the bombesin receptor family. After 24 h, cells were further incubated in serum-free media and GRP/NMB peptides (0/10/100/1,000 nM) or the N-terminal peptides of mBRS3 (1,000 nM) with different concentrations for another 12 h. Luciferase activities were determined using luciferase assay kits (Beyotime, Shanghai, China) and normalized to β-galactosidase activity. All experiments were performed at least three times in triplicates. HEK293 cells transfected with pcDNA were used as a blank control in all luciferase experiments, and the fold was calculated compared to blank control.

### ERK1/2 phosphorylation

For ERK1/2 phosphorylation, HEK293 cells were seeded in 24-well plates and transfected with GRPR, NMBR, BRS3, mutants of different species’ BRS3, or an empty vector plasmid, pcDNA3.1-V5-His plasmid (300 ng). After 36 h, cells were incubated in serum-free media for another 8 h; stimulated with 1,000 nm GRP or NMB for 0, 2, and 5 min or 0, 2, 5, and 10 min; homogenized in lysis buffer containing 50 mM Tris-HCl (pH 6.8) and 2% sodium dodecyl sulphate (SDS) with freshly added protease/phosphatase inhibitor cocktail (Cell Signaling Technology, Indianapolis, IN, USA); and subjected to western blot using specific antibodies for ERK1/2 (Cell Signaling Technology, Cat. #9102, 1:2,000) and phosphor-ERK1/2 (Cell Signaling Technology, Cat. #9101, 1:1,000). Each sample with an equal amount of protein was mixed with 6×SDS sample buffer, boiled for 5 min, and separated on 10% SDS-polyacrylamide gel electrophoresis (PAGE) before transferring the proteins onto a PVDF membrane. The membranes were then blocked at room temperature for 1 h with 5% milk powder in Tris-buffered saline-Tween (TBST), followed by subsequent incubation at 4°C overnight in TBST containing the different primary antibodies (1:1,000 dilution). After washing three times (10 min each time) with TBST, the membranes were incubated for 1 h in TBST containing the secondary antibodies (1:2,000 dilution), followed by washing three times (10 min each time) with TBST, prior to detection by chemiluminescence.

### Expression pattern of BRS3 receptors and their mutants

HEK293 cells were seeded in 24-well plates, and the expression vector pcDNA3.1-V5-His containing the different BRS3 orthologs or relevant mutants was transiently transfected into HEK 293 cells using Lipofectamine 2000 (Invitrogen) with 300 ng of different species’ BRS3 receptor or mutants. After 48 h, supernatant was removed and cells rinsed twice with PBS, homogenized in lysis buffer, and subjected to western blot analysis using specific Bombesin Receptor Polyclonal Antibody (Thermo Fisher, Cat. #PA5-26484/Lot. #A81B02N, 1:1,000) and actin as a control (Sigma-Aldrich, A5441, 1:5,000).

### Intracellular calcium assay

Intracellular calcium was measured using the non-wash calcium assay Fluo8 kit (ab112129, Abcam) according to the manufacturer’s instructions. Briefly, HEK293 cells were seeded in 24-well plates and were transfected with GRPR, NMBR, BRS3, or the empty vector plasmid pcDNA3.1-V5-His (300 ng) and incubated overnight at 37°C with 5% CO_**2**_. The next day, the cells were replated into a 96-well assay plate (black plate and clear bottom). After 12 h, growth media were aspirated, and calcium dye was added. Following incubation for 30 min at 37°C and 10 min at room temperature, GRP (10 nM) or NMB (10 nM) was added, and assay plates were placed into a fluorescence kinetic plate reader (Hamamatsu) immediately. The basal fluorescence intensity was recorded 15 times at 1 Hz for 20 s. The results were normalized to the average basal fluorescence intensity in ratio, and the peak response was used for the result calculation. Calcium fold was calculated using no stimulation data as standard value.

### Radioligand binding assays

The unlabeled ligand peptides GRP (APLQPGGSPALTKIYPRGSHWAVGHLM) and NMB (YKIKVNPRGNLWATGHFM) were synthesized by [^**125**^I]-Tyr^**42**^-GRP and [^**125**^I]-Tyr-NMB (Anhui Guoping Pharmaceutical) at a specific activity of 870 Ci/mmol and 690 Ci/mmol, respectively, and were prepared by the Beijing North Institute of Biotechnology. HEK293 cells were seeded in a 10-cm tissue culture dish at a density of 10^**6**^ cells per dish and grown overnight at 37°C in growth medium. The following morning, 5 μg of different species’ BRS3 plasmids were transfected. After 6 h, the medium was replaced with growth medium. Cells were maintained at 37°C in a 5% CO_**2**_ atmosphere and used 48 h later for binding assays. The positive control group included transfected GRPR and NMBR; the test group consisted of transfected BRS3 from different species. Then, [^**125**^I]-Tyr^**42**^-GRP and [^**125**^I]-Tyr-NMB were used as the ligands to assess affinity to the various receptors. Briefly, 48 h after transient transfection with Lipofectamine, disaggregated transfected cells were incubated for 1 h at 21°C in 250 μl of binding buffer containing 24.5 mM HEPES (pH 7.4), 98 mM NaCl, 6 mM KCl, 2.5 mM KH_**2**_PO_**4**_, 5 mM sodium pyruvate, 5 mM sodium fumarate, 5 mM sodium glutamate, 2 mM glutamine, 11.5 mM glucose, 0.5 mM CaCl_**2**_, 1 mM MgCl_**2**_, 0.01% (w/v) soybean trypsin inhibitor, 0.2% (v/v) amino acid mixture, 0.2% (w/v) BSA, and 0.05% (w/v) bacitracin with 50 pM of [^**125**^I]-Tyr^**42**^-GRP (870 Ci/mmol) or 50 pM of [^**125**^I]-Tyr-NMB (690 Ci/mmol) in the presence of the indicated concentration of unlabeled peptides. The cell concentration was adjusted to between 0.05 and 4 × 10^**6**^ cells/ml for each receptor such that less than 20% of the total added radioactive ligand was bound during the incubation, and the results were compared to cells transfected with GRPR or NMBR adjusted in concentration to bind a similar amount of ligand. After the incubation, 100 μl aliquots were added to 1.5-ml microfuge tubes, which contained 100 μl of binding buffer to determine the total radioactivity. The bound tracer was separated from unbound tracer by pelleting the cells through the binding buffer by centrifugation at 10,000*g* in a Microfuge E (Beckman) for 3 min. The supernatant was aspirated, and the pelleted cells were rinsed twice with a washing buffer that contained 1% (w/v) BSA in PBS. The amount of radioactivity bound to the cells was measured in a Cobra II Gamma counter (Packard Instruments). Binding was expressed as the percentage of total radioactivity that was associated with the cell pellet. All binding values represented saturable binding; nonsaturable binding was <15% of the total binding in all experiments. Each point was measured in duplicate, and each experiment was replicated at least four times. Calculation of affinity was performed by determining the IC_**50**_ (the GRP or NMB concentration causing half-maximum inhibition of binding), using the curve-fitting program KaleidaGraph (Synergy Software), and the Hill coefficient (nH) was calculated from the displacement curve by using GraphPad Prism [78]. All the experiment data were calculated using 1 pM ligand-treated as 100% control.

### The prediction of binding pocket of Gs, G12, and GPCR

Discovery Studio is a suite of packages for simulating macromolecule systems [37]. The largest possible binding pocket of Gs, G12, and GPCR was then predicted by Discovery Studio 3.0 [37]. These predicted pockets were utilized to construct an initial coarse model of the G protein–GPCR complex. The molecular model of GPCR receptors and G protein were predicted by the I-TASSER algorithm. I-TASSER was designed for protein structure modeling by iterative threading assembly simulations [27]. Starting from an amino acid sequence, I-TASSER first generates 3D atomic models from multiple threading alignments and iterative structural assembly simulations. The function of the protein is then inferred by structurally matching the 3D models with other known proteins. The output from a typical server run contains full-length secondary and tertiary structure predictions and functional annotations on ligand-binding sites, Enzyme Commission numbers, and Gene Ontology terms. An estimate of accuracy of the predictions is provided based on the confidence score of the modeling. Previous study revealed the existence of a selectivity barcode (that is, patterns of amino acids) on each of the 16 human G proteins, which is recognized by distinct regions on the approximately 800 human receptors [1]. Therefore, the docking between GPCRs and G proteins was explored based on the known R-G protein co-crystal structures and homology modeling. The docking process between G protein and GPCRs was the same as the docking process between BRS3 receptors and GPR/NMB. The model with the lowest energy was then obtained using Rosetta software (RosettaDock and FelxPepDock module) [38]. All types of noncovalent interactions at the atomic level in a protein structure could be identified by the RING (http://protein.bio.unipd.it/ring/) [40]. Binding sites between proteins in complex were then obtained by RING.

### cAMP assay

HEK293 cells were cultured as a monolayer on culture plates to 80%–90% confluency. Cells were harvested and centrifuged twice at 1,000 rpm for 5 min. The amount of cAMP produced was determined with the cAMP ELISA Detection Kit (GenScript). Three thousand cells per well were preincubated for 45 min at 37°C and subsequently at room temperature for 3 h with a range of agonist concentrations. The incubation was stopped by adding detection mix and antibody solution, according to the instructions of the manufacturer. The generated fluorescence intensity was then quantified finally with Synergy H1(BioTek).

### GTPgamma35S incorporation assay

Assays were run in 20 mM HEPES, 100 mM NaCl, 8 mM MgCl_**2**_, and 10 μg·mL^**–1**^ at pH 7.4 in a final volume of 200 μl in 1.5-ml tubes at 25°C. One hundred microliters of membrane preparation (20 μg protein per well) containing 5 μM GDP was added, followed by the addition of 10 μl of buffer agonists being tested and 10 μl of GTPgamma35S to provide a final concentration in the assay of 400 pM. Cell membranes were incubated for 30 min at 25°C with agonists, followed by addition of GTPgamma35S and incubation for an additional 60 min. Preincubation was employed to ensure that agonists were at equilibrium during the labeling period. Then, 35S-labeled membranes were solubilized for 30 min with 0.27% Nonidet P-40, followed by the addition of the desired antibody (10 μl/well) to provide a final dilution of 1/200 and incubation for an additional 60 min. Fifty microliters of suspended anti-IgG-coated SPA beads was added per tube. Tubes were incubated for 3 h and then were centrifuged, and their radioactivity was determined using a Aloka LSC-8000 counter.

### Statistical analysis

Experiments were repeated independently at least three times. Results were analyzed using GraphPad Prism 5. Differences between two groups were compared using two-tailed Student *t* test. One-way ANOVA was followed by a Fisher’s LSD post hoc test to evaluate the differences among multiple groups. Data are expressed as mean ± SEM. Calculations were done with a standard statistical package (SPSS for Windows, version 21). Statistical significance was defined as a *P* value < 0.05 (*) or *P* value < 0.01(**) [79].

## Supporting information

S1 Table Species list of bombesin receptors and out-group. **+** represents the corresponding amino acid sequence of the species that was selected for phylogenetic analysis. The species are as follows: *Homo*: *H*. *sapiens*; *Pan*: *P*. *troglodytes*; *Mus*: *M*. *musculus*; *Rattus*: *R*. *norvegicus*; *Sus*: *S*. *scrofa*; *Capra*: *C*. *hircus*; *Ovis*: *O*. *aries*; *Canis*: *C*. *lupus familiaris*; *Felis*: *F*. *catus*; *Orycteropus*: *O*. *afer*; *Loxodonta*: *L*. *africana*; *Phascolarctos*: *P*. *cinereus*; *Monodelphis*: *M*. *domestica*; *Ornithorhynchus*: *O*. *anatinus*; *Gallus*: *G*. *gallus*; *Anolis*; *A*. *carolinensis*; *Chrysemys*: *C*. *picta*; *Xenopus*: *X*. *tropicalis*; *Lepisosteus*: *L*. *oculatus*; *Danio*: *D*. *rerio*; *Saccoglossus*: *S*. *kowalevskii*; *Acanthaster*: *A*. *planci*; *Strongylocentrotus*: *S*. *purpuratus*; *Apis*: *A*. *mellifera*; *Nasonia*: *N*. *vitripennis*; *Drosophila*: *D*. *melanogaster*; *Aedes*: *A*. *aegypti*; *Tribolium*: *T*. *castaneum*; *Camponotus*: *C*. *floridanus*; *Parasteatoda*: *P*. *tepidariorum*; *Myzus*: *M*. *persicae*; *Lingula*: *L*. *anatina*; *Crassostrea*: *C*. *virginica*; and *Mizuhopecten*: *M*. *yessoensis*.
(TIF)

S2 Table Barcodes of placental mammalian BRS3 receptor binding G protein. Red font indicates amino acid sites for mutation, which exhibited a large degree of conservation in placental mammals, and the sites differs from the corresponding amino acids in nonplacental species. BRS3, bombesin receptor subtype-3.
(XLSX)

S3 Table Potential binding sites of nonplacental vertebrate BRS3 receptors with GRP or NMB peptides. BRS3, bombesin receptor subtype-3; GRP, gastrin-releasing peptide; NMB, neuromedin B.
(XLSX)

S1 Fig Comparison of RMSDs and structural similarity of BRS3 between placental mammals and nonplacental vertebrates. (A) Comparison of sequence identity and sequence similarity of placental mammalian and nonplacental vertebrate BRS3 to GRPRs/NMBRs, respectively. The y-axis indicates the value obtained by comparing BRS3 with GRPRs/NMBRs. Sequence identity, sequence similarity, and RMSD in placental mammals were shown in red frame. The species are as follows: *Homo*: *H*. *sapiens*; *Pan*: *P*. *troglodytes*; *Mus*: *M*. *musculus*; *Rattus*: *R*. *norvegicus*; *Canis*: *C*. *lupus familiaris*; *Felis*: *F*. *catus*; *Orycteropus*: *O*. *afer*; *Loxodonta*: *L*. *africana*; *Phascolarctos*: *P*. *cinereus*; *Monodelphis*: *M*. *domestica*; *Ornithorhynchus*: *O*. *anatinus*; *Gallus*: *G*. *gallus*; *Corvus*: *Corvus brachyrhynchos*; *Anolis*: *A*. *carolinensis*; *Chrysemys*: *C*. *picta*; *Xenopus*: *X*. *tropicalis*; and *Lepisosteus*: *L*. *oculatus*. (B) Structural similarity of supplementary species BRS3 with GRPRs. Red, gray, and blue represent placental mammalian BRS3, nonplacental vertebrate BRS3, and GRPR, respectively. (C) Structural similarity of supplementary species BRS3 with NMBRs. Red, gray, and cyan represent placental mammalian BRS3, nonplacental vertebrate BRS3, and NMBR, respectively. The underlying data can be found in S6 Data. BRS3, bombesin receptor subtype-3; GRPR, gastrin-releasing peptide receptor; NMBR, neuromedin B receptor; RMSD, root-mean-square deviation.
(TIF)

S2 Fig Comparison of similarity between in GRPRs and NMBRs. (A) Comparison of sequence and structural similarity of GRPRs to NMBRs. The triangle, square, and circle represent structural similarity, sequence similarity, and sequence identity. The y-axis indicates the value obtained by comparing GRPRs and NMBRs. The species are as follows: *Homo*: *H*. *sapiens*; *Pan*: *P*. *troglodytes*; *Mus*: *M*. *musculus*; *Rattus*: *R*. *norvegicus*; *Canis*: *C*. *lupus familiaris*; *Felis*: *F*. *catus*; *Orycteropus*: *O*. *afer*; *Loxodonta*: *L*. *africana*; *Phascolarctos*: *P*. *cinereus*; *Monodelphis*: *M*. *domestica*; *Ornithorhynchus*: *O*. *anatinus*; *Gallus*: *G*. *gallus*; *Corvus*: *C*. *brachyrhynchos*; *Anolis*: *A*. *carolinensis*; *Chrysemys*: *C*. *picta*; *Xenopus*: *X*. *tropicalis*; and *Lepisosteus*: *L*. *oculatus*. (B) Structural similarity of supplementary species GRPR with NMBRs. Blue and cyan represent GRPR and NMBR. The underlying data can be found in S7 Data. GRPR, gastrin-releasing peptide receptor; NMBR, neuromedin B receptor.
(TIF)

S3 Fig Sequence alignment of GRP (top) and NMB peptides (bottom). The red box indicates the highly conserved areas, which is a mature peptide we used for all functional tests except for radioligand binding assays. The species are as follows: *Homo*: *H*. *sapiens*; *Pan*: *P*. *troglodytes*; *Mus*: *M*. *musculus*; *Rattus*: *R*. *norvegicus*; *Dipodomys*: *Dipodomys ordii*; *Sus*: *S*. *scrofa*; *Capra*: *C*. *hircus*; *Ovis*: *O*. *aries*; *Canis*: *C*. *lupus familiaris*; *Felis*: *F*. *catus*; *Gallus*: *G*. *gallus*; *Corvus*: *C*. *brachyrhynchos*; *Anolis*: *A*. *carolinensis*; *Chrysemys*: *C*. *picta*; *Xenopus*: *X*. *tropicalis*; *Lepisosteus*: *L*. *oculatus*. GRP, gastrin-releasing peptide; NMB, neuromedin B.
(TIF)

S4 Fig Different evolution, different protein structures, and different function between nonplacental vertebrate BRS3 and placental mammalian BRS3. The left lane-phylogenetic tree represents evolution of 17 vertebrate species; red and bold lines represent adaptive evolution in placental mammals. The species are as follows: 17 vertebrate species (8 placental mammals: *Homo* and *Pan* represent Euarchonta, *Mus* and *Rattus* represent Glires, *Canis* and *Felis* represent Laurasiatheria, and *Orycteropus* and *Loxodonta* represent Atlantogenata; 3 nonplacental mammals: *Phascolarctos* and *Monodelphis* represent Marsupialia, and *Ornithorhynchus* represents Monotremata; 6 nonmammalian vertebrates: *Gallus* and *Corvus* represent bird, *Chrysemys* and *Anolis* represent reptile, *Xenopus* represents amphibian, and *Lepisosteus* represents fish). The lanes for SI, SS, and RMSDs represent comparison of sequence and structural similarity of placental mammalian and nonplacental vertebrate BRS3. Red and black represent placental mammalian BRS3 and nonplacental vertebrate BRS3 receptors, respectively. The triangle, square, and circle represent structural similarity–RMSD, SS, and SI, respectively. The y-axis in RMSD lane indicates the average value obtained by comparing BRS3 with NMBRs/GRPRs. Ten representative vertebrate species were selected for structure and function analysis—4 placental mammals: human/*Homo* represents Euarchonta, mouse/*Mus* represents Glires, dog/*Canis* represents Laurasiatheria, and aardvark/*Orycteropus* represents Atlantogenata; 2 nonplacental mammals: koala/*Phascolarctos* represents Marsupialia, and platypus/*Ornithorhynchus* represents Monotremata; 4 nonmammalian vertebrates: chicken/*Gallus* represents bird, turtle/*Chrysemys* represents reptile, frog/*Xenopus* represents amphibian, and spotted gar/*Lepisosteus* represents fish. The structure lane represents the overlap of 10 representative vertebrate BRS3 with NMBRs/GRPRs. Red, gray, cyan, and blue represent placental mammalian BRS3, nonplacental vertebrate BRS3, NMBR, and GRPR, respectively. The ERK lane: the phosphorylation levels of ERK for each of BRS3 receptors. GRP and NMB peptides are utilized to activate BRS3 in placental mammals and nonplacental vertebrates, respectively. Three time points of 0, 2, and 5 min were chosen. The calcium lane: the levels of Ca^2+^ ions in cells for each of the BRS3 receptors. The luciferase lane: constitutive activity for BRS3 in placental mammals but not in nonplacental vertebrates. The bottom lane: the negative control and positive control of NMB-NMBR and GRP-GRPR for stimulation of Ca^2+^ ions and phosphorylation levels of ERK. The underlying data can be found in S8 Data BRS3, bombesin receptor subtype-3; ERK, extracellular signal–regulated kinase; GRP, gastrin-releasing peptide receptor; GRPR, GRP receptor; NMB, neuromedin B receptor; NMBR, NMB receptor; pERK, phosphorylated ERK; RMSD, root-mean-square deviation; SI, sequence identity; SS, sequence similarity.
(TIF)

S5 Fig Quantification of ERK and intracellular calcium activated by GRP and NMB peptides between nonplacental vertebrate BRS3 and placental mammalian BRS3. This figure is supplementary data for Fig S4. (A) The phosphorylation levels of ERK for each mutant of BRS3 receptors. GRP and NMB peptides are utilized to activate BRS3 in placental mammals and nonplacental vertebrates, respectively. Three time points of 0, 2, and 5 min were chosen. ERK was calculated by comparing the pERK value to the ERK value. (B) The levels of Ca^2+^ ions in cells for each mutant of the BRS3 receptors. GRP and NMB peptides are utilized to activate BRS3 receptors respectively. The calcium fold is calculated by fluorescence intensity (excitation/emission wavelength: 490/520 nm). The underlying data can be found in S9 Data BRS3, bombesin receptor subtype-3; ERK, extracellular signal–regulated kinase; GRP, gastrin-releasing peptide receptor; GRPR, GRP receptor; NMB, neuromedin B receptor; NMBR, NMB receptor; pERK, phosphorylated ERK.
(TIF)

S6 Fig BRS3 in placental mammals and nonplacental vertebrates activate Gs, Gq, G12, and Gi signaling with NMB/GRP or not. (A) BRS3 from 10 vertebrate species was tested for G protein signaling using CRE, NFAT, SRF, and SRE luciferase assay. (B) BRS3 in placental mammals cannot activate Gq signaling in a ligand (GRP: upper; NMB: lower) in a dose-dependent manner. The underlying data can be found in S10 Data. aBRS3, aardvark BRS3; BRS3, bombesin receptor subtype-3; cBRS3, chicken BRS3; CRE, cAMP response element; dBRS3, dog BRS3; fBRS3, frog BRS3; GRP, gastrin-releasing peptide; hBRS3, human BRS3; kBRS3, koala BRS3; mBRS3, mouse BRS3; NFAT, nuclear factor of activated T cells; NMB, neuromedin B; pBRS3, platypus BRS3; sBRS3, spotted gar BRS3; SRE, serum response element; SRF, serum response factor; tBRS3, turtle BRS3.
(TIF)

S7 Fig Expression levels of the BRS3 receptor protein of various species and the corresponding mutants in the HEK293 cell line. (A-C) The primary antibodies are BRS3 and actin, and the molecular weights are 36 kDa and 42 kDa, respectively. All plasmids were transfected with same amounts except in panel B; in panel B, we used the same protein concentration for this western blot and in vivo BRS3 from the mouse brain and in vitro BRS3 from transfected HEK293 cells with different doses of mBRS3 plasmid. (D) The primary antibodies are anti-HA tag antibody, and the molecular weights are about 46 kD for both NMBR and GRPR. BRS3, bombesin receptor subtype-3; HA, hemagglutinin; HEK293, human embryonic kidney 293; mBRS3, mouse BRS3; NMBR, neuromedin B receptor.
(TIF)

S8 Fig mBRS3 increases cAMP accumulation in HEK293 cells. Fold was calculated using HEK293 cells transfected with pcDNA as control. The underlying data can be found in S11 Data. HEK293, human embryonic kidney 293; mBRS3, mouse bombesin receptor subtype-3.
(TIF)

S9 Fig NMB inhibits GRPR–GRP binding with low affinity. The underlying data can be found in S12 Data. GRP, gastrin-releasing peptide; GRPR, GRP receptor; NMB, neuromedin B.
(TIF)

S10 Fig Sotrastaurin inhibited the Gq downstream signaling mediated by BRS3, NMBR, and GRPR. (A) Sotrastaurin inhibits the phosphorylation levels of ERK for BRS3 receptors in nonplacental vertebrates. Receptor-expressing HEK293 cells were pretreated with inhibitor for 1 h with 25 μM. Subsequently, GRP or NMB peptides were added to the cells at a concentration of 1 μM for 5 min before western blot. (B) Sotrastaurin inhibits Ca^2+^ ion levels in cells transfected with BRS3 receptors of nonplacental vertebrates. Receptor-expressing HEK293 cells were pretreated with inhibitor for 1 h with 25 μM. Subsequently, GRP or NMB were added to the cells at a concentration of 10 nM prior to the assay. The calcium fold is calculated by fluorescence intensity (excitation/emission wavelength: 490/520 nm). (C) Sotrastaurin inhibits the constitutively activated Gq signaling pathway with BRS3 of placental mammals. Receptor-expressing HEK293 cells were pretreated with 25 μM inhibitor for 12 h prior to the luciferase assays. The underlying data can be found in S13 Data. BRS3, bombesin receptor subtype-3; ERK, extracellular signal–regulated kinase; GRP, gastrin-releasing peptide; GRPR, GRP receptor; HEK293, human embryonic kidney 293; NMB, neuromedin B; NMBR, NMB receptor.
(TIF)

S11 Fig Important residues on the sequence logo of BRS3 receptors in placental mammals (black) and nonplacental vertebrates (blue). The green, red, and purple triangles indicate positive selection sites for the N terminus of placental mammalian BRS3, potential key interaction sites of nonplacental vertebrate BRS3, and mutation sites of action with G protein, respectively. BRS3, bombesin receptor subtype-3.
(TIF)

S12 Fig Sequence alignment of BRS3 receptors in placental mammals and nonplacental vertebrates. The green, red, and purple frames indicate positive selection sites for the N terminus of placental mammalian BRS3, key binding/activating sites of BRS3 in nonplacental vertebrates for recognition of GRP and NMB, and barcodes of placental mammalian BRS3 receptor–binding G protein, respectively. BRS3, bombesin receptor subtype-3; GRP, gastrin-releasing peptide; NMB, neuromedin B.
(TIF)

S13 Fig Mutants of barcode-neighboring residues of mBRS3 activate Gs and G12 signaling. (A) Constitutive activity for each mutants of barcode-neighboring residues of mBRS3. Statistical significance was defined as a *P* value < 0.05 (*). (B) Expression levels for mutants of barcode-neighboring residues of mBRS3. In panel B, * represents lower expression than in the WT. Mutation of barcode-neighboring residues has no effect or little effect except for reduced expression on the BRS3 receptor activity. The underlying data can be found in S14 Data. BRS3, bombesin receptor subtype-3; mBRS3, mouse BRS3; WT, wild type.
(TIF)

S14 Fig BRS3 in site-mutative placental mammals and nonplacental vertebrates activate Gs, Gq, G12, and Gi signaling with NMB/GRP. (A) BRS3 in site-mutative species was tested for G protein signaling with NMB peptides using CRE, NFAT, SRF, and SRE luciferase assay. (B) BRS3 in site-mutative species was tested for G protein signaling with GRP peptides using CRE, NFAT, SRF, and SRE luciferase assay. The underlying data can be found in S15 Data. aBRS3, aardvark BRS3; BRS3, bombesin receptor subtype-3; cBRS3, chicken BRS3; CRE, cAMP response element; GRP, gastrin-releasing peptide; hBRS3, human BRS3; mBRS3, mouse BRS3; NFAT, nuclear factor of activated T cells; NMB, neuromedin B; sBRS3, spotted gar BRS3; SRE, serum response element; SRF, serum response factor; tBRS3, turtle BRS3.
(TIF)

S15 Fig The constitutive signaling activation of triple mutations of placental mammalian and nonplacental vertebrate BRS3. The underlying data can be found in S16 Data. BRS3, bombesin receptor subtype-3.
(TIF)

S16 Fig The N-terminal truncated BRS3 in nonplacental vertebrates (cBRS3, sBRS3, and tBRS3) cannot be stimulated by N-terminal peptide. The underlying data can be found in S17 Data. BRS3, bombesin receptor subtype-3; cBRS3, chicken BRS3; spotted gar BRS3; tBRS3, turtle BRS3.
(TIF)

S17 Fig Constitutive activity of placental mammalian BRS3 with N terminus or not. The constitutive activation level of truncated hBRS3, mBRS3, and aBRS3 receptors is significantly lower than intact ones in Gs, Gq, and G12 signaling. The underlying data can be found in S18 Data. aBRS3, aardvark BRS3; BRS3, bombesin receptor subtype-3; hBRS3, human BRS3; mBRS3, mouse BRS3.
(TIF)

S18 Fig Triple mutations in the N terminus of placental and nonplacental animal BRS3. The 14I, 23S, and 40N triple mutants in placental BRS3 changed the helix structure of the N terminus, whereas in nonplacental BRS3 they form the helix structure in the N terminus. BRS3, bombesin receptor subtype-3.
(TIF)

S1 Data (XLSX)

S2 Data (XLSX)

S3 Data (XLSX)

S4 Data (XLSX)

S5 Data (XLSX)

S6 Data (XLSX)

S7 Data (XLSX)

S8 Data (XLSX)

S9 Data (XLSX)

S10 Data (XLSX)

S11 Data (XLSX)

S12 Data (XLSX)

S13 Data (XLSX)

S14 Data (XLSX)

S15 Data (XLSX)

S16 Data (XLSX)

S17 Data (XLSX)

S18 Data (XLSX)

## Abbreviations

2R: two rounds of whole-genome duplications
7TM: seven-transmembrane
aBRS3: aardvark BRS3
BLP: bombesin-like peptide
BRS3: bombesin receptor subtype-3
cBRS3: chicken BRS3
CCHaR-1: CCHamide-1 receptor
CCHaR-2: CCHamide-2 receptor
CNS: central nervous system
CPM: counts per minute
df: degree of freedom
ENDRA: endothelin receptor type A
ERK: extracellular signal–regulated kinase
CRE: cAMP response element
GPCR: G protein–coupled receptor
GRP: gastrin-releasing peptide
GRPR: GRP receptor
hBRS3: human BRS3
HEK293: human embryonic kidney 293
I-TASSER: Iterative Threading Assembly Refinement
LRT: likelihood ration test
mBRS3: mouse BRS3
NFAT-RE: nuclear factor of activated T cells response element
NMB: neuromedin B
NMBR: NMB receptor
NA: not available
NS: not significant
PAML: phylogenetic analysis by maximum likelihood
pERK: phosphorylated ERK
RING: Residue Interaction Network Generator
RMSD: root-mean-square deviation
sBRS3: spotted gar BRS3
SRE: serum response element
SRF-RE: serum response factor response element
tBRS3: turtle BRS3
