# Supplementary figures and images for: Constitutively active BRS3 is a genuinely orphan GPCR in placental mammals
Source: PLoS Biol. 2019 Mar 6;17(3):e3000175. doi: 10.1371/journal.pbio.3000175 (PMC6422423; doi:10.1371/journal.pbio.3000175)

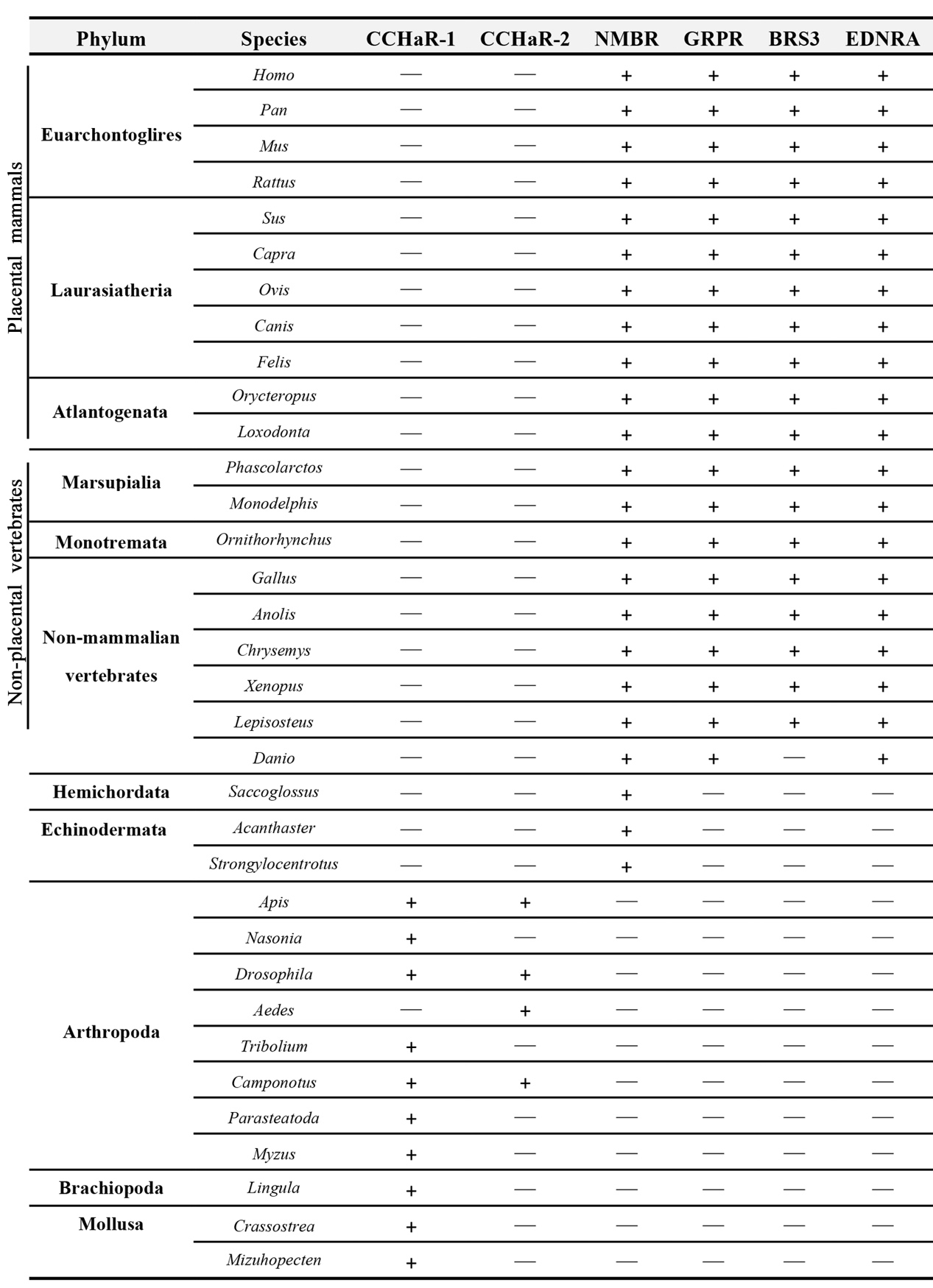

Supplement: S1 Table — + represents the corresponding amino acid sequence of the species that was selected for phylogenetic analysis. The species are as follows: Homo: H. sapiens; Pan: P. troglodytes; Mus: M. musculus; Rattus: R. norvegicus; Sus: S. scrofa; Capra: C. hircus; Ovis: O. aries; Canis: C. lupus familiaris; Felis: F. catus; Orycteropus: O. afer; Loxodonta: L. africana; Phascolarctos: P. cinereus; Monodelphis: M. domestica; Ornithorhynchus: O. anatinus; Gallus: G. gallus; Anolis; A. carolinensis; Chrysemys: C. picta; Xenopus: X. tropicalis; Lepisosteus: L. oculatus; Danio: D. rerio; Saccoglossus: S. kowalevskii; Acanthaster: A. planci; Strongylocentrotus: S. purpuratus; Apis: A. mellifera; Nasonia: N. vitripennis; Drosophila: D. melanogaster; Aedes: A. aegypti; Tribolium: T. castaneum; Camponotus: C. floridanus; Parasteatoda: P. tepidariorum; Myzus: M. persicae; Lingula: L. anatina; Crassostrea: C. virginica; and Mizuhopecten: M. yessoensis. (TIF) [file pbio.3000175.s001.tif]

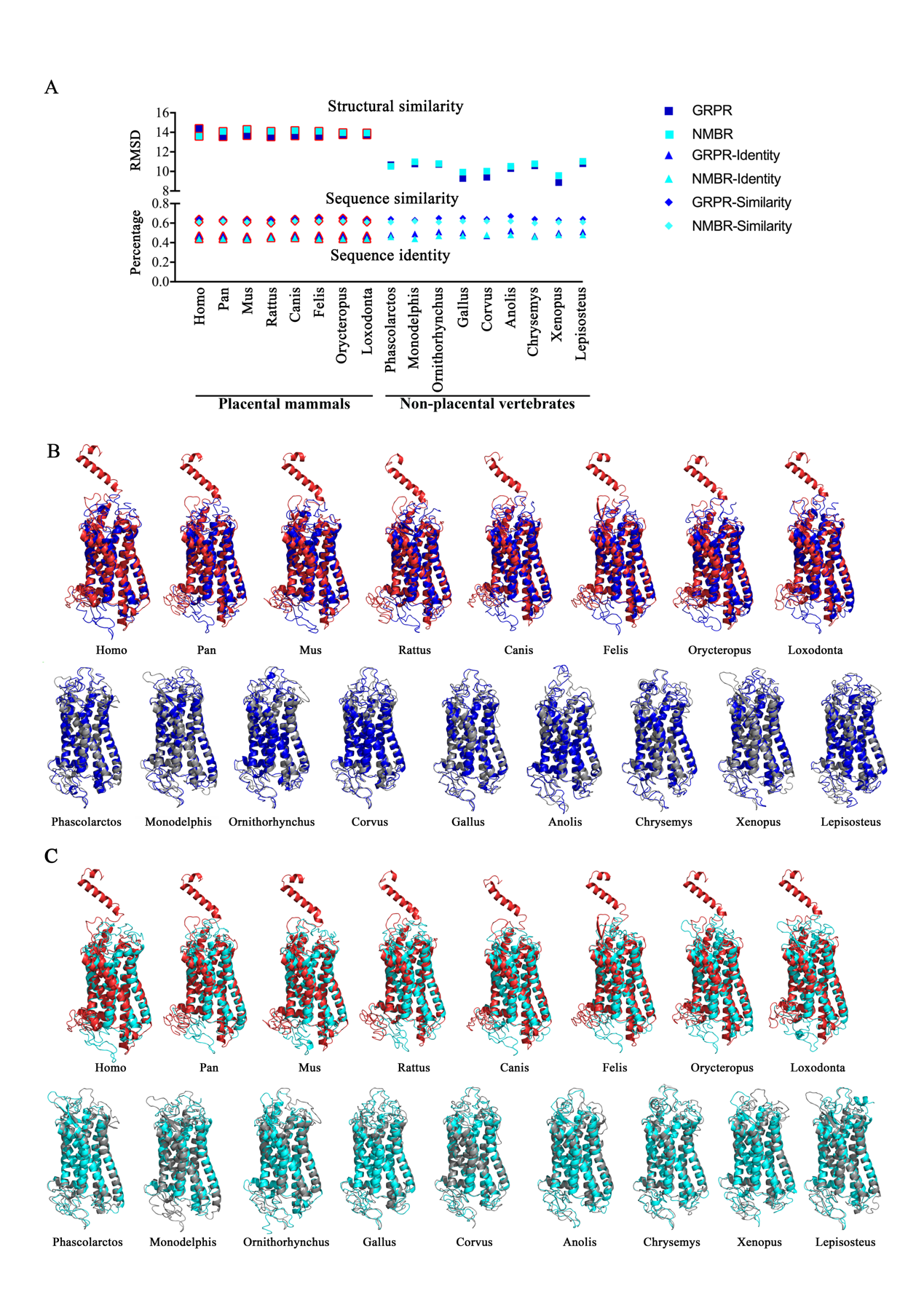

Supplement: S1 Fig — (A) Comparison of sequence identity and sequence similarity of placental mammalian and nonplacental vertebrate BRS3 to GRPRs/NMBRs, respectively. The y-axis indicates the value obtained by comparing BRS3 with GRPRs/NMBRs. Sequence identity, sequence similarity, and RMSD in placental mammals were shown in red frame. The species are as follows: Homo: H. sapiens; Pan: P. troglodytes; Mus: M. musculus; Rattus: R. norvegicus; Canis: C. lupus familiaris; Felis: F. catus; Orycteropus: O. afer; Loxodonta: L. africana; Phascolarctos: P. cinereus; Monodelphis: M. domestica; Ornithorhynchus: O. anatinus; Gallus: G. gallus; Corvus: Corvus brachyrhynchos; Anolis: A. carolinensis; Chrysemys: C. picta; Xenopus: X. tropicalis; and Lepisosteus: L. oculatus. (B) Structural similarity of supplementary species BRS3 with GRPRs. Red, gray, and blue represent placental mammalian BRS3, nonplacental vertebrate BRS3, and GRPR, respectively. (C) Structural similarity of supplementary species BRS3 with NMBRs. Red, gray, and cyan represent placental mammalian BRS3, nonplacental vertebrate BRS3, and NMBR, respectively. The underlying data can be found in S6 Data. BRS3, bombesin receptor subtype-3; GRPR, gastrin-releasing peptide receptor; NMBR, neuromedin B receptor; RMSD, root-mean-square deviation. (TIF) [file pbio.3000175.s004.tif]

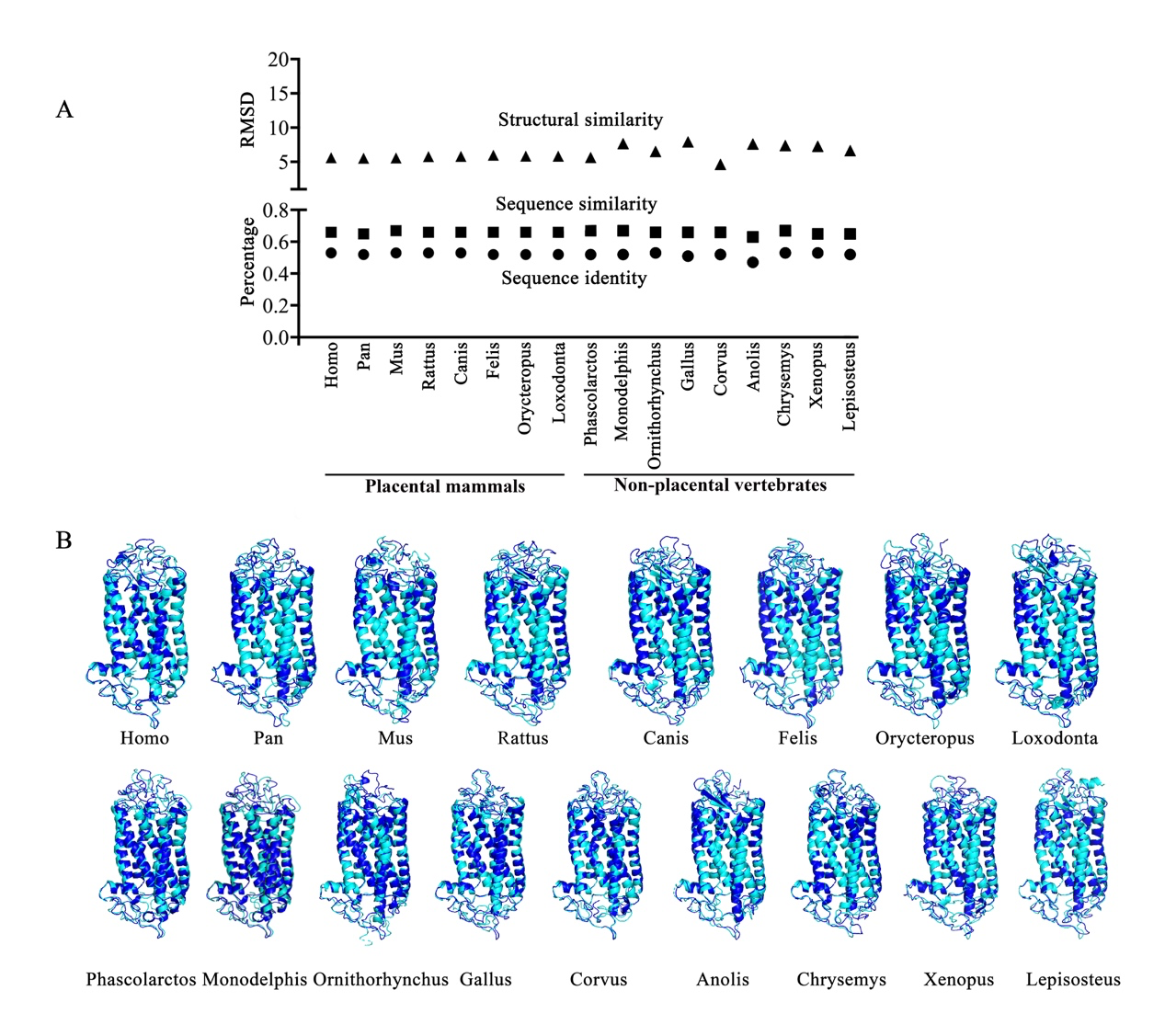

Supplement: S2 Fig — (A) Comparison of sequence and structural similarity of GRPRs to NMBRs. The triangle, square, and circle represent structural similarity, sequence similarity, and sequence identity. The y-axis indicates the value obtained by comparing GRPRs and NMBRs. The species are as follows: Homo: H. sapiens; Pan: P. troglodytes; Mus: M. musculus; Rattus: R. norvegicus; Canis: C. lupus familiaris; Felis: F. catus; Orycteropus: O. afer; Loxodonta: L. africana; Phascolarctos: P. cinereus; Monodelphis: M. domestica; Ornithorhynchus: O. anatinus; Gallus: G. gallus; Corvus: C. brachyrhynchos; Anolis: A. carolinensis; Chrysemys: C. picta; Xenopus: X. tropicalis; and Lepisosteus: L. oculatus. (B) Structural similarity of supplementary species GRPR with NMBRs. Blue and cyan represent GRPR and NMBR. The underlying data can be found in S7 Data. GRPR, gastrin-releasing peptide receptor; NMBR, neuromedin B receptor. (TIF) [file pbio.3000175.s005.tif]

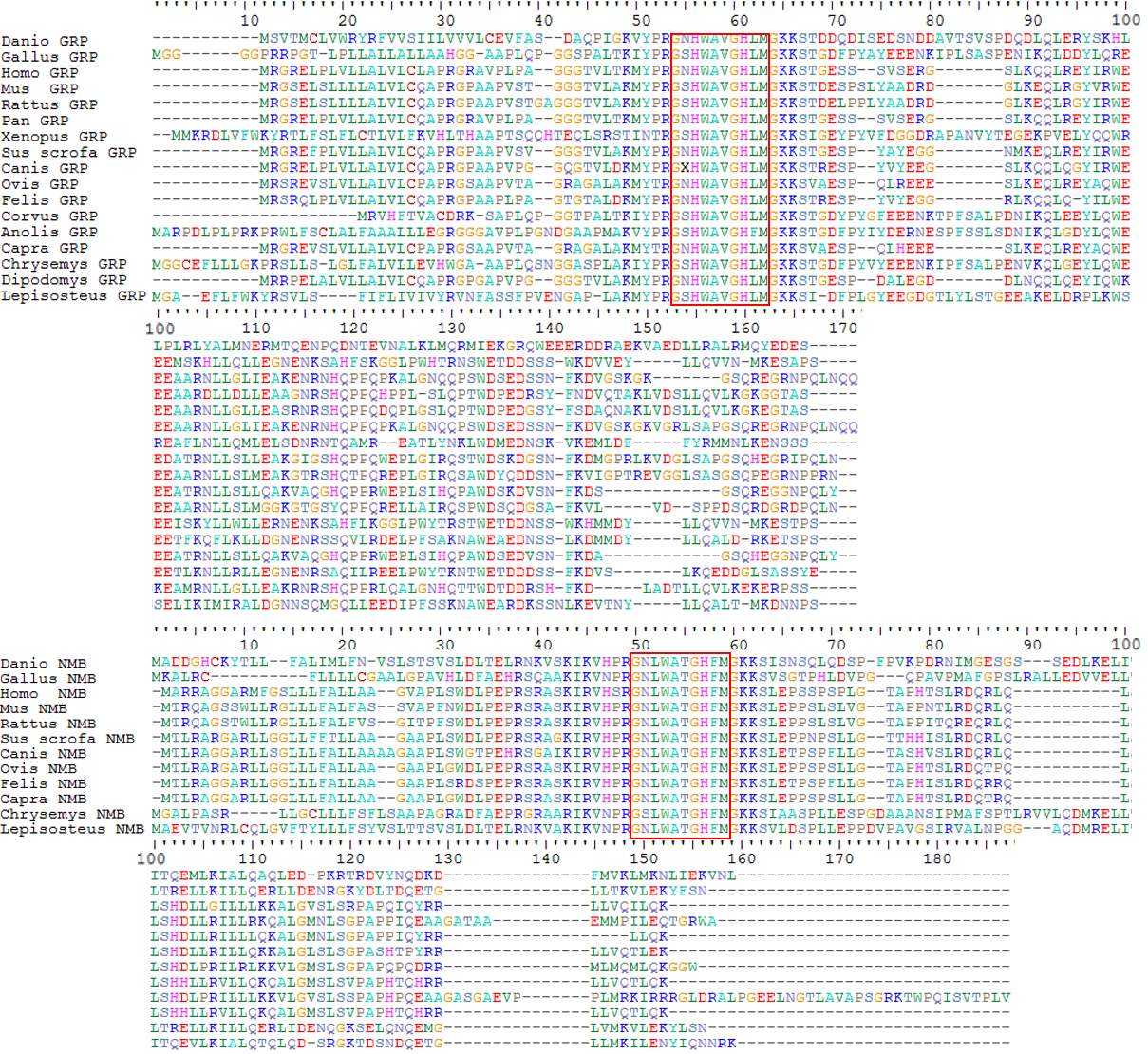

Supplement: S3 Fig — The red box indicates the highly conserved areas, which is a mature peptide we used for all functional tests except for radioligand binding assays. The species are as follows: Homo: H. sapiens; Pan: P. troglodytes; Mus: M. musculus; Rattus: R. norvegicus; Dipodomys: Dipodomys ordii; Sus: S. scrofa; Capra: C. hircus; Ovis: O. aries; Canis: C. lupus familiaris; Felis: F. catus; Gallus: G. gallus; Corvus: C. brachyrhynchos; Anolis: A. carolinensis; Chrysemys: C. picta; Xenopus: X. tropicalis; Lepisosteus: L. oculatus. GRP, gastrin-releasing peptide; NMB, neuromedin B. (TIF) [file pbio.3000175.s006.tif]

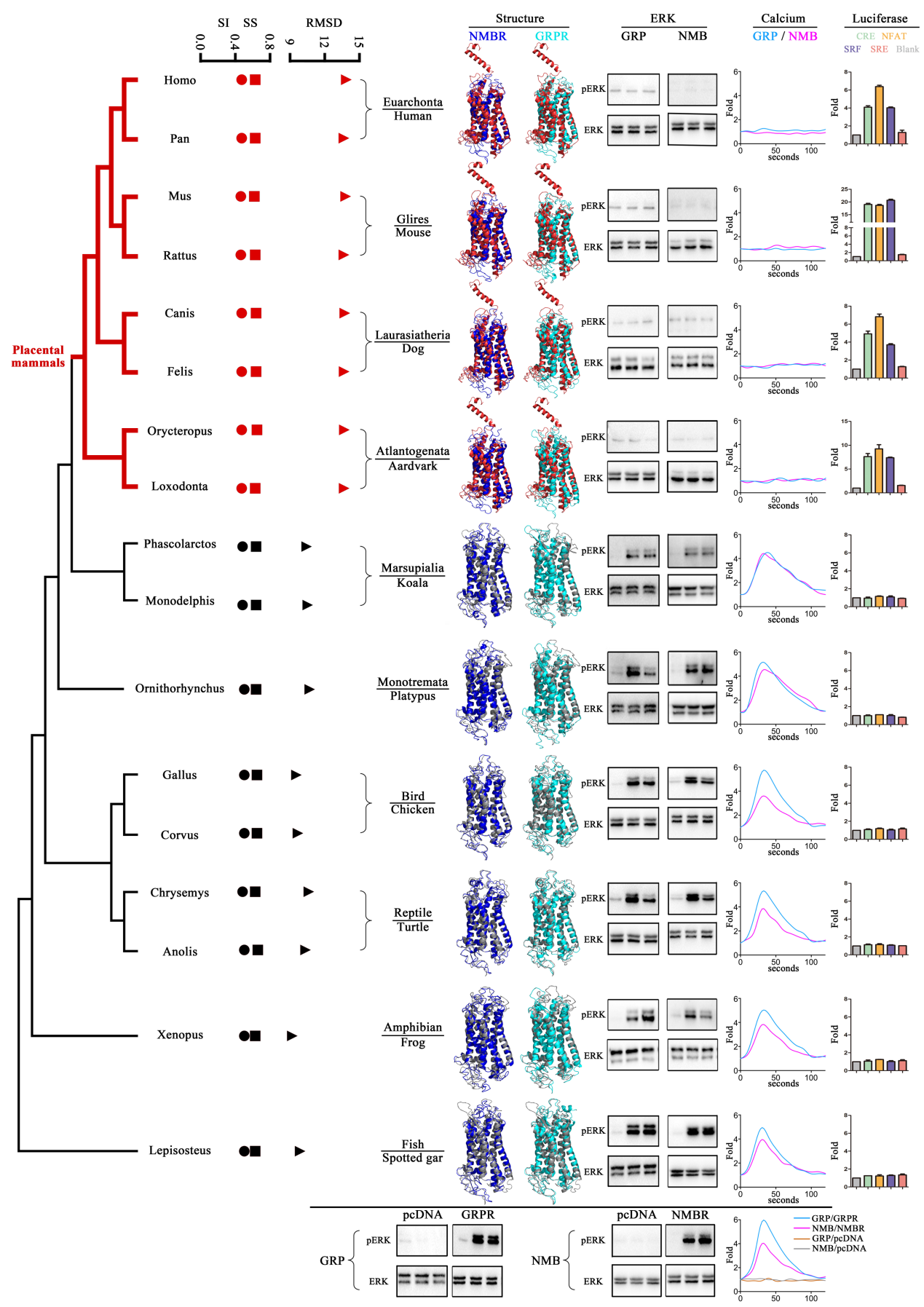

Supplement: S4 Fig — The left lane-phylogenetic tree represents evolution of 17 vertebrate species; red and bold lines represent adaptive evolution in placental mammals. The species are as follows: 17 vertebrate species (8 placental mammals: Homo and Pan represent Euarchonta, Mus and Rattus represent Glires, Canis and Felis represent Laurasiatheria, and Orycteropus and Loxodonta represent Atlantogenata; 3 nonplacental mammals: Phascolarctos and Monodelphis represent Marsupialia, and Ornithorhynchus represents Monotremata; 6 nonmammalian vertebrates: Gallus and Corvus represent bird, Chrysemys and Anolis represent reptile, Xenopus represents amphibian, and Lepisosteus represents fish). The lanes for SI, SS, and RMSDs represent comparison of sequence and structural similarity of placental mammalian and nonplacental vertebrate BRS3. Red and black represent placental mammalian BRS3 and nonplacental vertebrate BRS3 receptors, respectively. The triangle, square, and circle represent structural similarity–RMSD, SS, and SI, respectively. The y-axis in RMSD lane indicates the average value obtained by comparing BRS3 with NMBRs/GRPRs. Ten representative vertebrate species were selected for structure and function analysis—4 placental mammals: human/Homo represents Euarchonta, mouse/Mus represents Glires, dog/Canis represents Laurasiatheria, and aardvark/Orycteropus represents Atlantogenata; 2 nonplacental mammals: koala/Phascolarctos represents Marsupialia, and platypus/Ornithorhynchus represents Monotremata; 4 nonmammalian vertebrates: chicken/Gallus represents bird, turtle/Chrysemys represents reptile, frog/Xenopus represents amphibian, and spotted gar/Lepisosteus represents fish. The structure lane represents the overlap of 10 representative vertebrate BRS3 with NMBRs/GRPRs. Red, gray, cyan, and blue represent placental mammalian BRS3, nonplacental vertebrate BRS3, NMBR, and GRPR, respectively. The ERK lane: the phosphorylation levels of ERK for each of BRS3 receptors. GRP and NMB peptides are [file pbio.3000175.s007.tif]

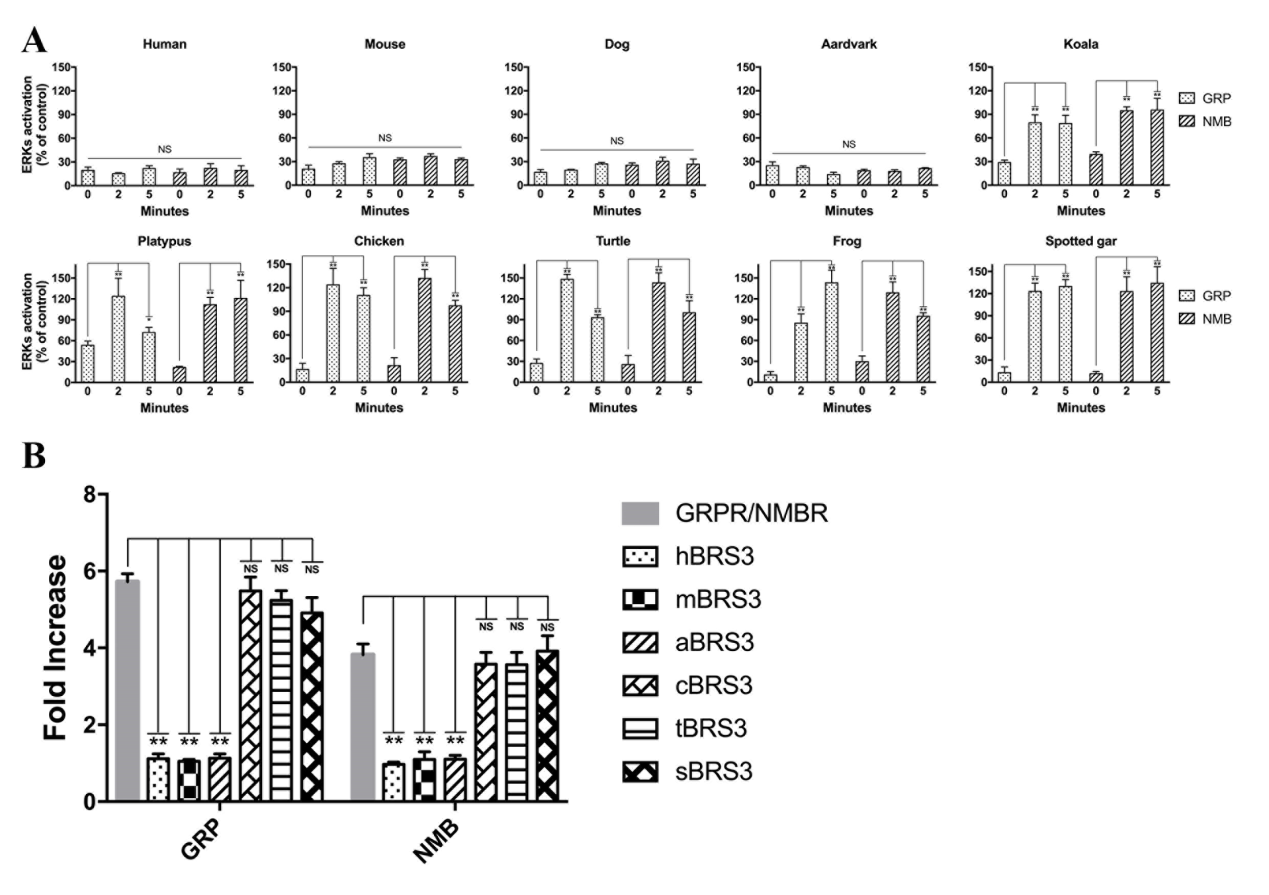

Supplement: S5 Fig — This figure is supplementary data for Fig S4. (A) The phosphorylation levels of ERK for each mutant of BRS3 receptors. GRP and NMB peptides are utilized to activate BRS3 in placental mammals and nonplacental vertebrates, respectively. Three time points of 0, 2, and 5 min were chosen. ERK was calculated by comparing the pERK value to the ERK value. (B) The levels of Ca2+ ions in cells for each mutant of the BRS3 receptors. GRP and NMB peptides are utilized to activate BRS3 receptors respectively. The calcium fold is calculated by fluorescence intensity (excitation/emission wavelength: 490/520 nm). The underlying data can be found in S9 Data BRS3, bombesin receptor subtype-3; ERK, extracellular signal–regulated kinase; GRP, gastrin-releasing peptide receptor; GRPR, GRP receptor; NMB, neuromedin B receptor; NMBR, NMB receptor; pERK, phosphorylated ERK. (TIF) [file pbio.3000175.s008.tif]

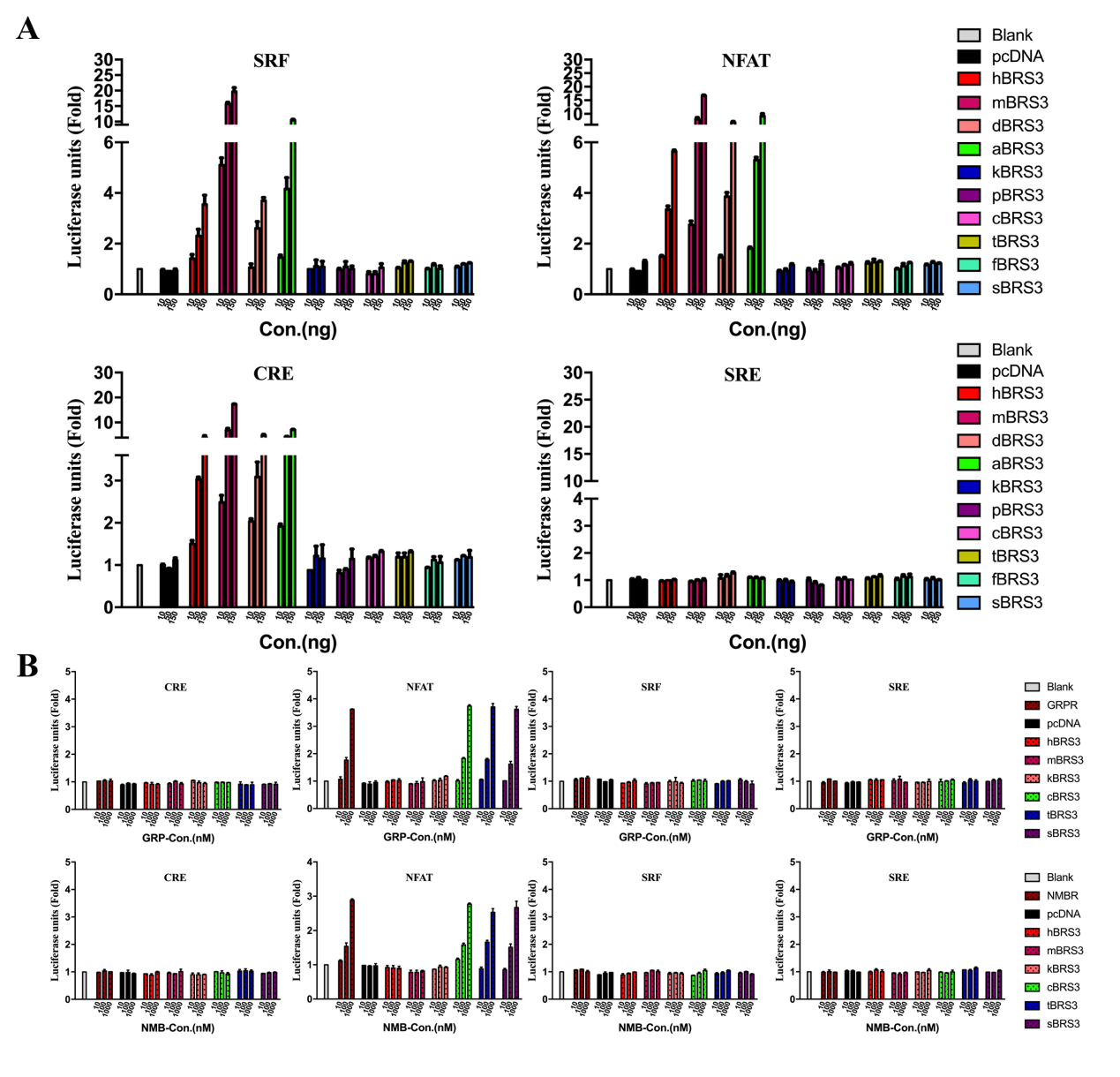

Supplement: S6 Fig — (A) BRS3 from 10 vertebrate species was tested for G protein signaling using CRE, NFAT, SRF, and SRE luciferase assay. (B) BRS3 in placental mammals cannot activate Gq signaling in a ligand (GRP: upper; NMB: lower) in a dose-dependent manner. The underlying data can be found in S10 Data. aBRS3, aardvark BRS3; BRS3, bombesin receptor subtype-3; cBRS3, chicken BRS3; CRE, cAMP response element; dBRS3, dog BRS3; fBRS3, frog BRS3; GRP, gastrin-releasing peptide; hBRS3, human BRS3; kBRS3, koala BRS3; mBRS3, mouse BRS3; NFAT, nuclear factor of activated T cells; NMB, neuromedin B; pBRS3, platypus BRS3; sBRS3, spotted gar BRS3; SRE, serum response element; SRF, serum response factor; tBRS3, turtle BRS3. (TIF) [file pbio.3000175.s009.tif]

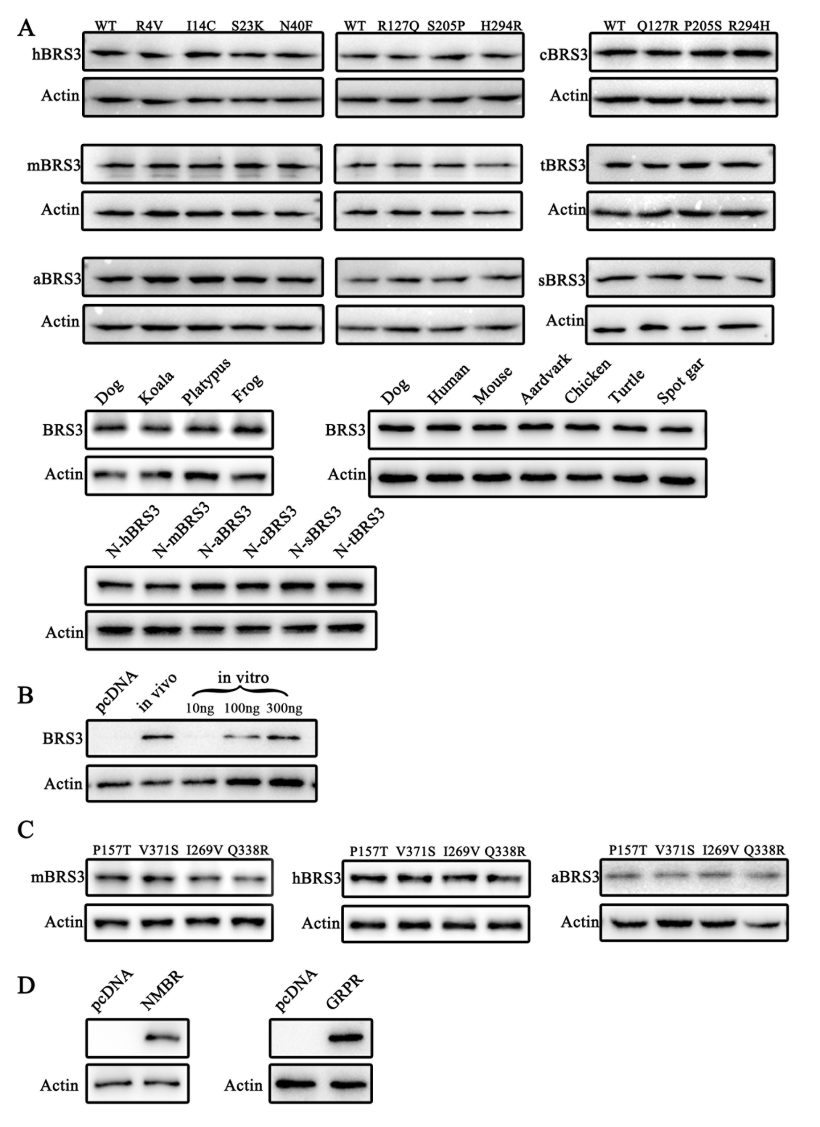

Supplement: S7 Fig — (A-C) The primary antibodies are BRS3 and actin, and the molecular weights are 36 kDa and 42 kDa, respectively. All plasmids were transfected with same amounts except in panel B; in panel B, we used the same protein concentration for this western blot and in vivo BRS3 from the mouse brain and in vitro BRS3 from transfected HEK293 cells with different doses of mBRS3 plasmid. (D) The primary antibodies are anti-HA tag antibody, and the molecular weights are about 46 kD for both NMBR and GRPR. BRS3, bombesin receptor subtype-3; HA, hemagglutinin; HEK293, human embryonic kidney 293; mBRS3, mouse BRS3; NMBR, neuromedin B receptor. (TIF) [file pbio.3000175.s010.tif]

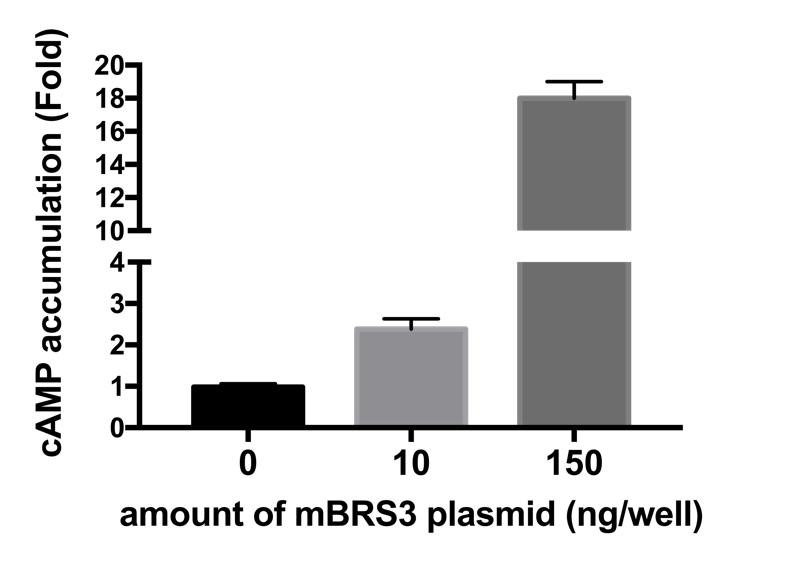

Supplement: S8 Fig — Fold was calculated using HEK293 cells transfected with pcDNA as control. The underlying data can be found in S11 Data. HEK293, human embryonic kidney 293; mBRS3, mouse bombesin receptor subtype-3. (TIF) [file pbio.3000175.s011.tif]

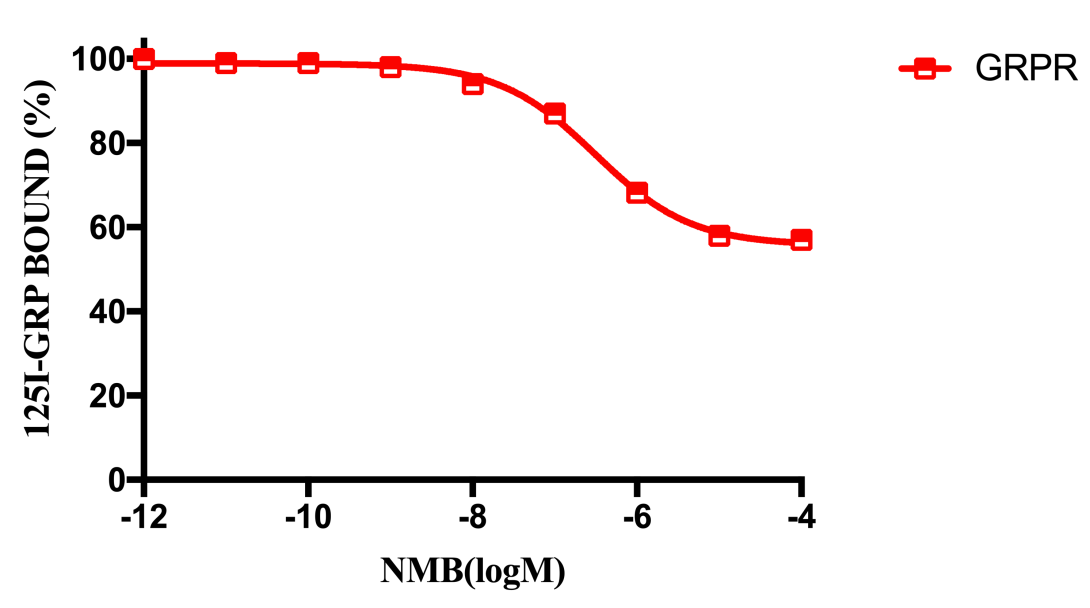

Supplement: S9 Fig — The underlying data can be found in S12 Data. GRP, gastrin-releasing peptide; GRPR, GRP receptor; NMB, neuromedin B. (TIF) [file pbio.3000175.s012.tif]

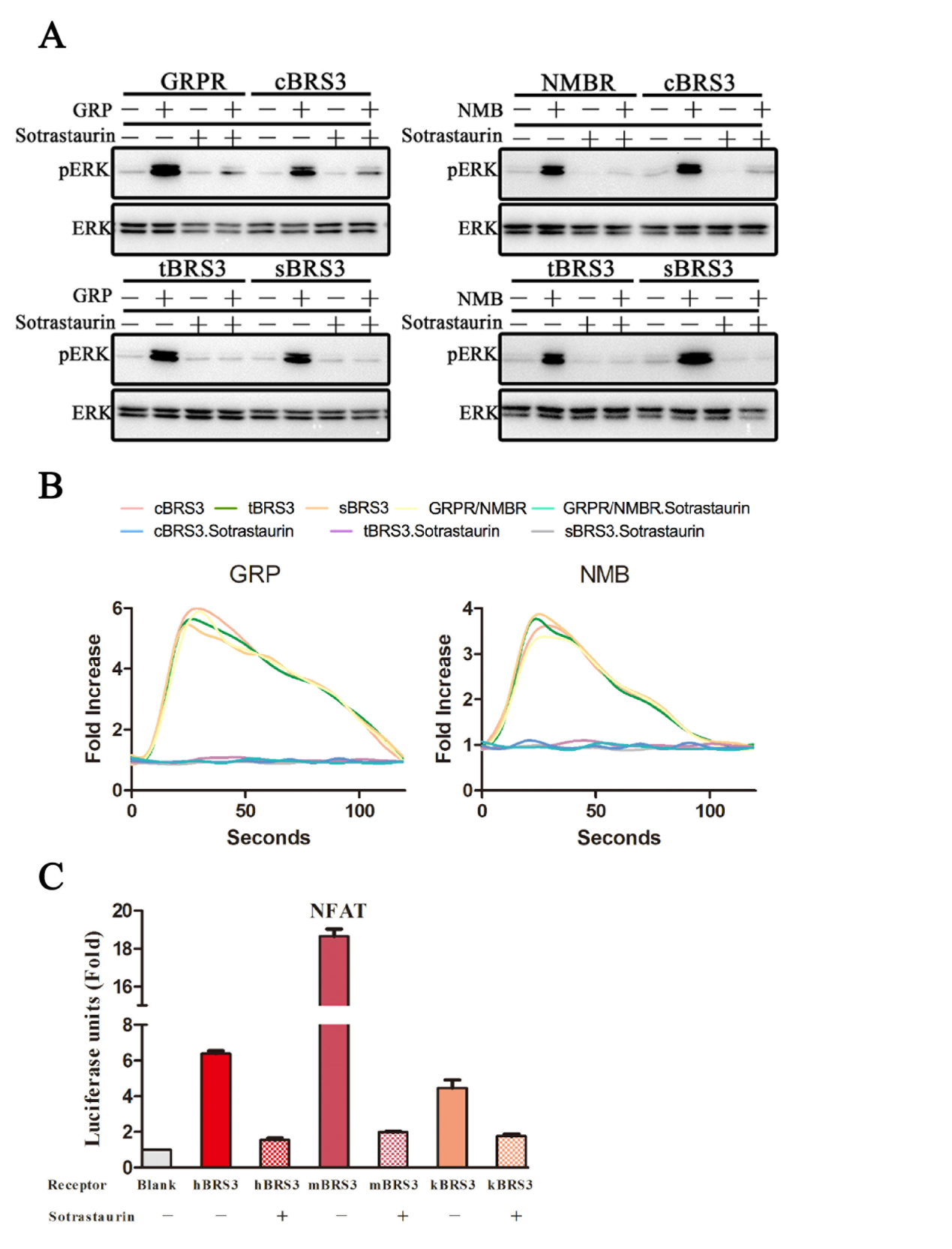

Supplement: S10 Fig — (A) Sotrastaurin inhibits the phosphorylation levels of ERK for BRS3 receptors in nonplacental vertebrates. Receptor-expressing HEK293 cells were pretreated with inhibitor for 1 h with 25 μM. Subsequently, GRP or NMB peptides were added to the cells at a concentration of 1 μM for 5 min before western blot. (B) Sotrastaurin inhibits Ca2+ ion levels in cells transfected with BRS3 receptors of nonplacental vertebrates. Receptor-expressing HEK293 cells were pretreated with inhibitor for 1 h with 25 μM. Subsequently, GRP or NMB were added to the cells at a concentration of 10 nM prior to the assay. The calcium fold is calculated by fluorescence intensity (excitation/emission wavelength: 490/520 nm). (C) Sotrastaurin inhibits the constitutively activated Gq signaling pathway with BRS3 of placental mammals. Receptor-expressing HEK293 cells were pretreated with 25 μM inhibitor for 12 h prior to the luciferase assays. The underlying data can be found in S13 Data. BRS3, bombesin receptor subtype-3; ERK, extracellular signal–regulated kinase; GRP, gastrin-releasing peptide; GRPR, GRP receptor; HEK293, human embryonic kidney 293; NMB, neuromedin B; NMBR, NMB receptor. (TIF) [file pbio.3000175.s013.tif]

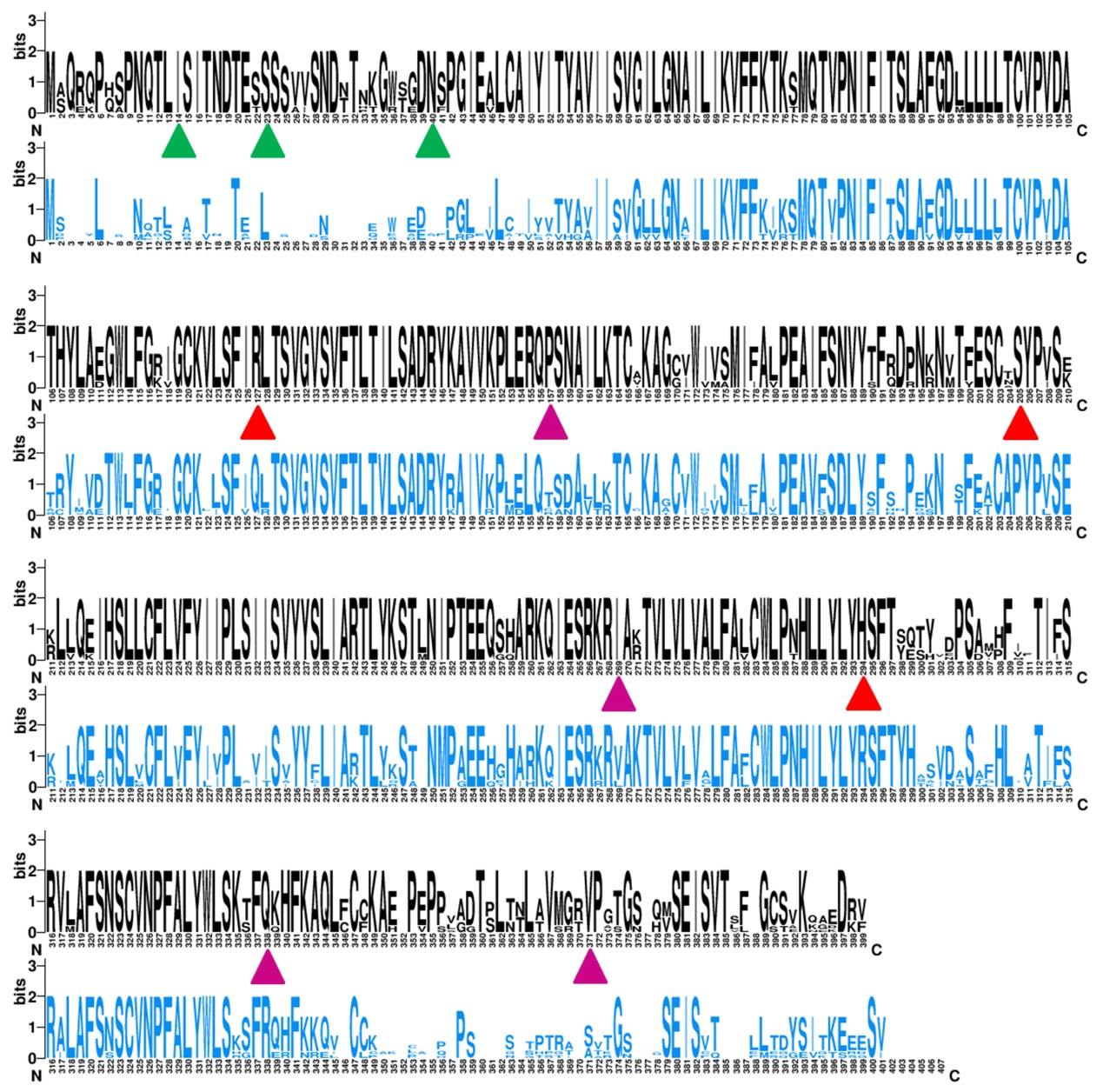

Supplement: S11 Fig — The green, red, and purple triangles indicate positive selection sites for the N terminus of placental mammalian BRS3, potential key interaction sites of nonplacental vertebrate BRS3, and mutation sites of action with G protein, respectively. BRS3, bombesin receptor subtype-3. (TIF) [file pbio.3000175.s014.tif]

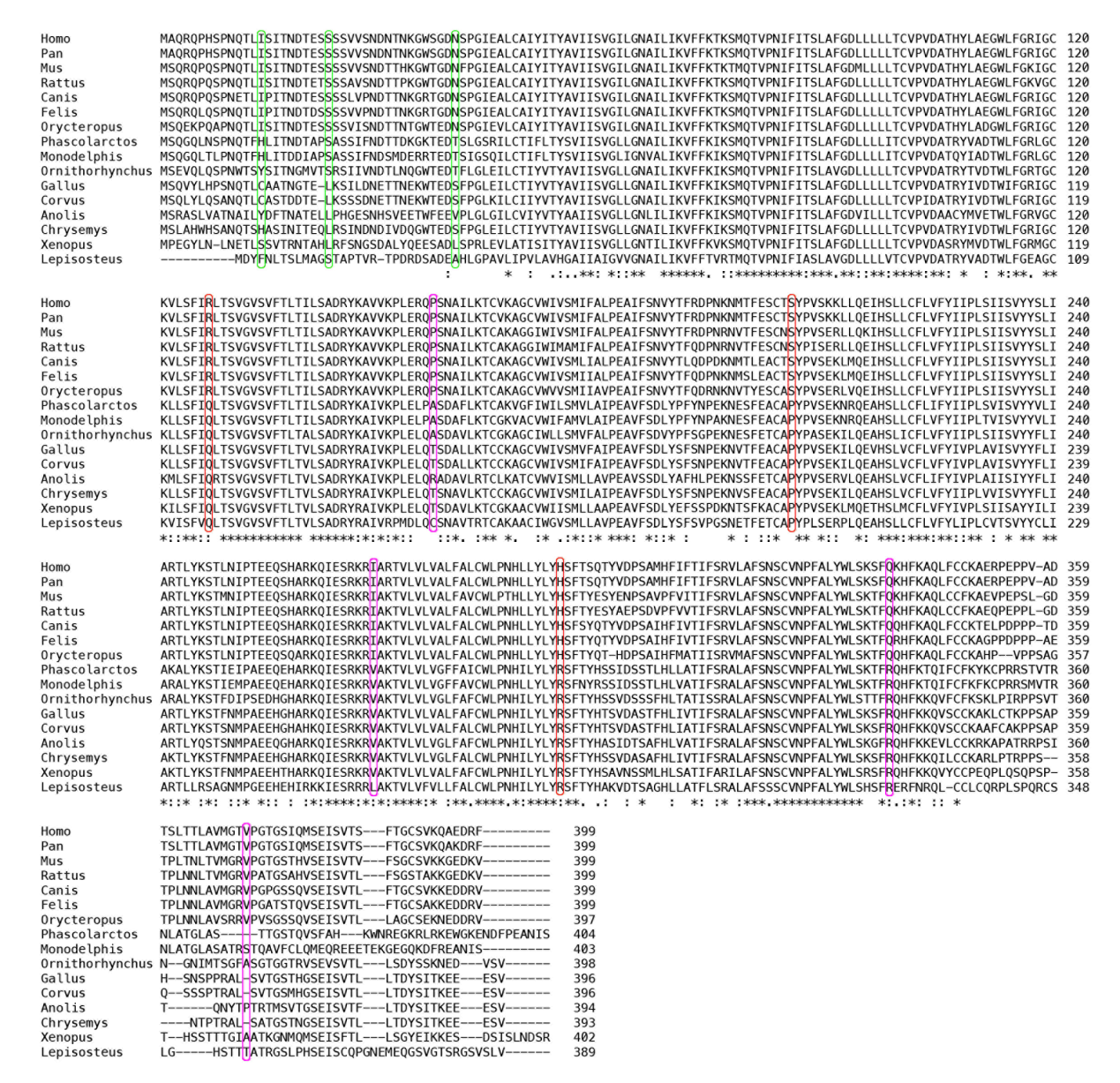

Supplement: S12 Fig — The green, red, and purple frames indicate positive selection sites for the N terminus of placental mammalian BRS3, key binding/activating sites of BRS3 in nonplacental vertebrates for recognition of GRP and NMB, and barcodes of placental mammalian BRS3 receptor–binding G protein, respectively. BRS3, bombesin receptor subtype-3; GRP, gastrin-releasing peptide; NMB, neuromedin B. (TIF) [file pbio.3000175.s015.tif]

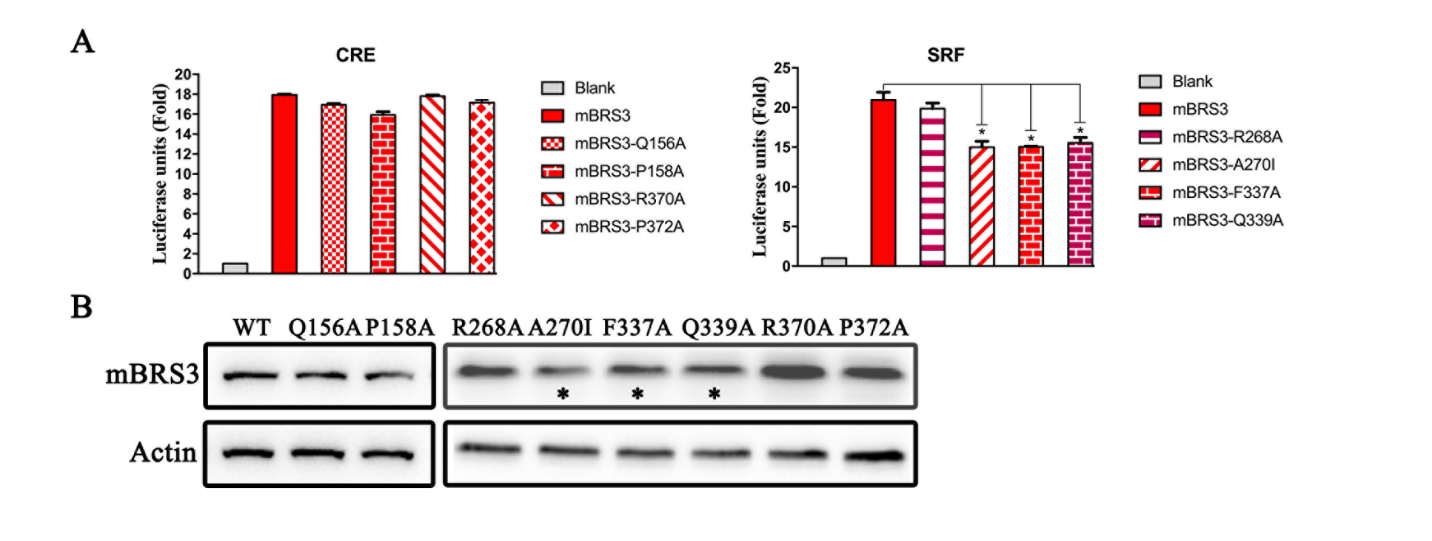

Supplement: S13 Fig — (A) Constitutive activity for each mutants of barcode-neighboring residues of mBRS3. Statistical significance was defined as a P value < 0.05 (*). (B) Expression levels for mutants of barcode-neighboring residues of mBRS3. In panel B, * represents lower expression than in the WT. Mutation of barcode-neighboring residues has no effect or little effect except for reduced expression on the BRS3 receptor activity. The underlying data can be found in S14 Data. BRS3, bombesin receptor subtype-3; mBRS3, mouse BRS3; WT, wild type. (TIF) [file pbio.3000175.s016.tif]

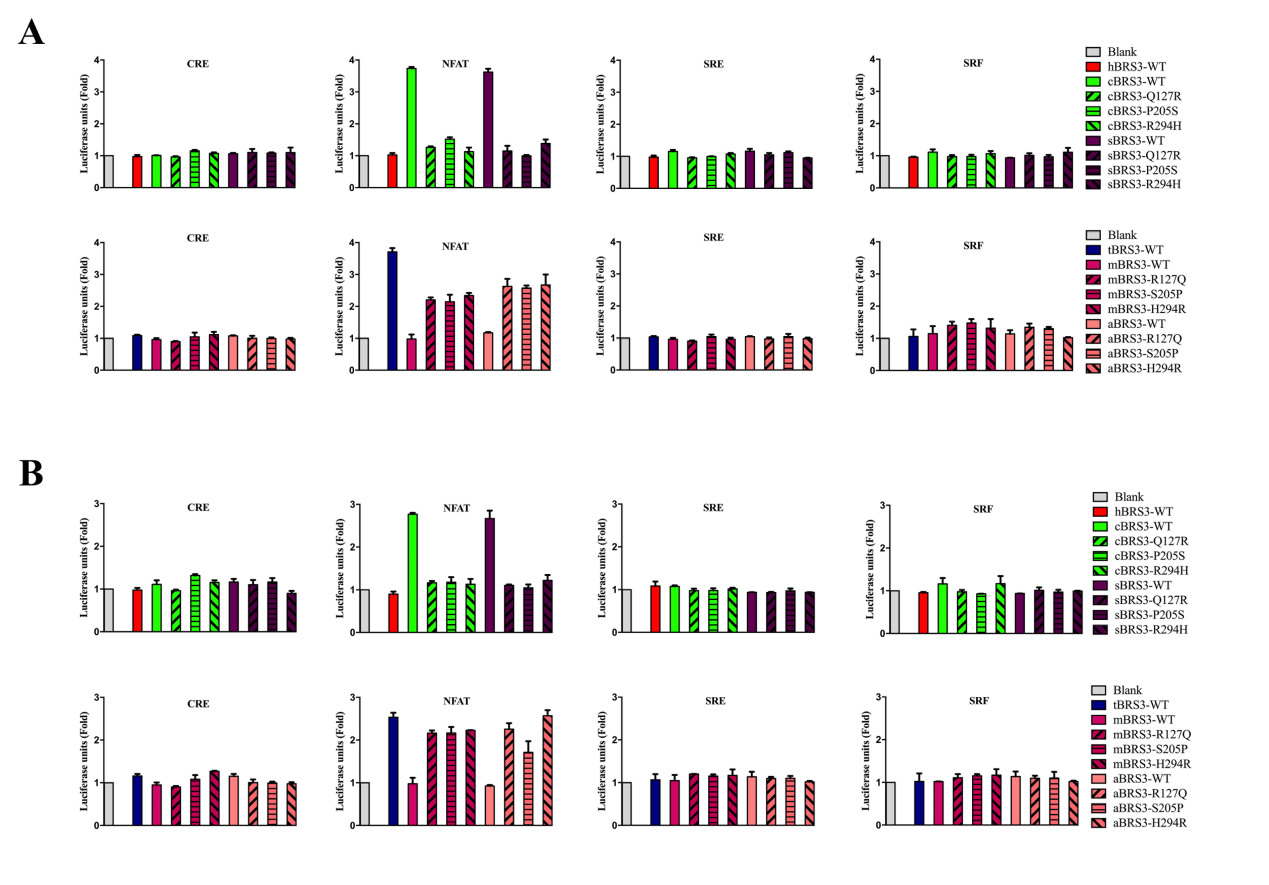

Supplement: S14 Fig — (A) BRS3 in site-mutative species was tested for G protein signaling with NMB peptides using CRE, NFAT, SRF, and SRE luciferase assay. (B) BRS3 in site-mutative species was tested for G protein signaling with GRP peptides using CRE, NFAT, SRF, and SRE luciferase assay. The underlying data can be found in S15 Data. aBRS3, aardvark BRS3; BRS3, bombesin receptor subtype-3; cBRS3, chicken BRS3; CRE, cAMP response element; GRP, gastrin-releasing peptide; hBRS3, human BRS3; mBRS3, mouse BRS3; NFAT, nuclear factor of activated T cells; NMB, neuromedin B; sBRS3, spotted gar BRS3; SRE, serum response element; SRF, serum response factor; tBRS3, turtle BRS3. (TIF) [file pbio.3000175.s017.tif]

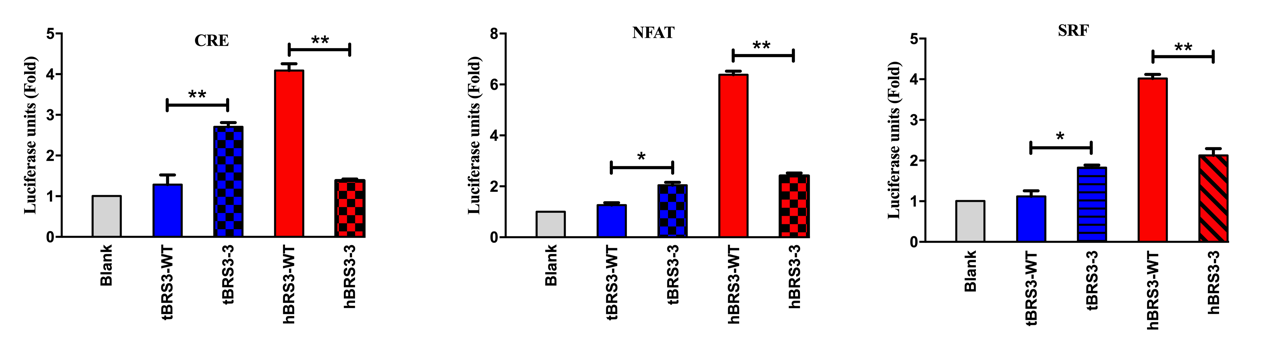

Supplement: S15 Fig — The underlying data can be found in S16 Data. BRS3, bombesin receptor subtype-3. (TIF) [file pbio.3000175.s018.tif]

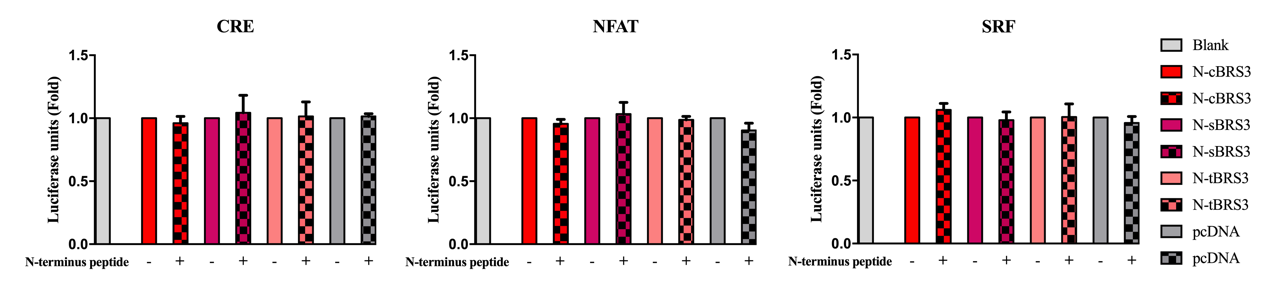

Supplement: S16 Fig — The underlying data can be found in S17 Data. BRS3, bombesin receptor subtype-3; cBRS3, chicken BRS3; spotted gar BRS3; tBRS3, turtle BRS3. (TIF) [file pbio.3000175.s019.tif]

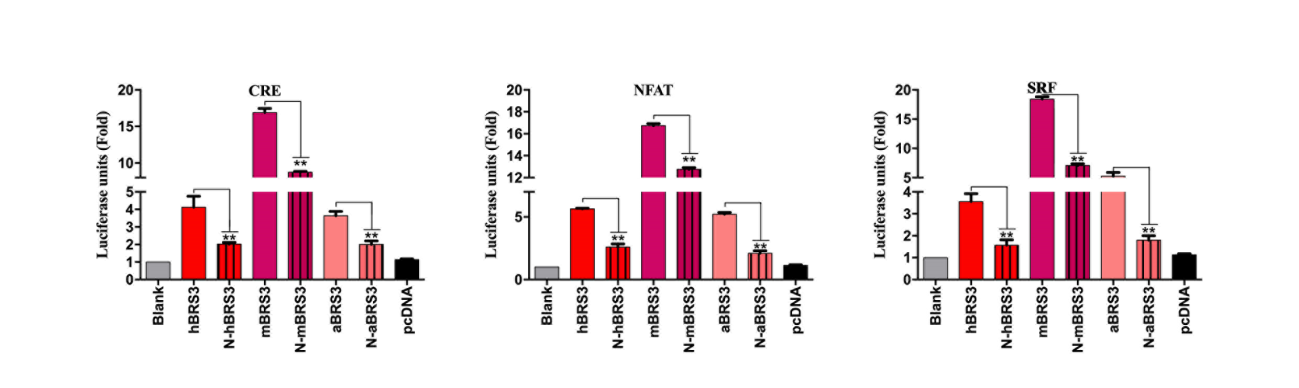

Supplement: S17 Fig — The constitutive activation level of truncated hBRS3, mBRS3, and aBRS3 receptors is significantly lower than intact ones in Gs, Gq, and G12 signaling. The underlying data can be found in S18 Data. aBRS3, aardvark BRS3; BRS3, bombesin receptor subtype-3; hBRS3, human BRS3; mBRS3, mouse BRS3. (TIF) [file pbio.3000175.s020.tif]

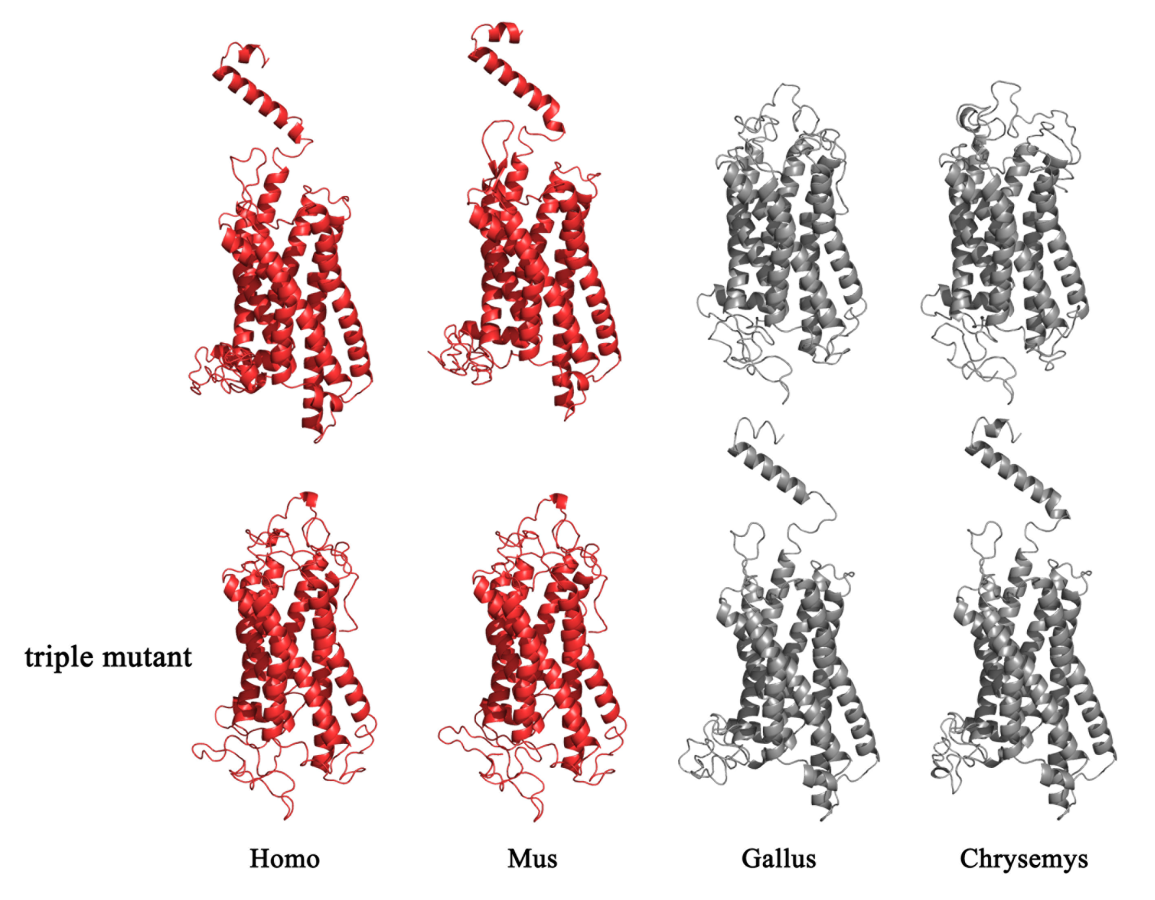

Supplement: S18 Fig — The 14I, 23S, and 40N triple mutants in placental BRS3 changed the helix structure of the N terminus, whereas in nonplacental BRS3 they form the helix structure in the N terminus. BRS3, bombesin receptor subtype-3. (TIF) [file pbio.3000175.s021.tif]
